# Monosaccharide-mediated rational synthesis of a universal plasmonic platform with broad spectral fluorescence enhancement for high-sensitivity cancer biomarker analysis

**DOI:** 10.1186/s12951-022-01359-z

**Published:** 2022-04-10

**Authors:** Mengyao Liu, Yonghong Li, Wei Xing, Yuqin Zhang, Xi Xie, Jiadong Pang, Fangjian Zhou, Jiang Yang

**Affiliations:** 1 State Key Laboratory of Oncology in South China, Collaborative Innovation Center for Cancer Medicine, Sun Yat-Sen University Cancer Center, Guangzhou, China; 2 Department of Urology, Sun Yat-Sen University Cancer Center, Guangzhou, China; 3 Department of Anesthesiology, Sun Yat-Sen University Cancer Center, Guangzhou, China; 4 School of Automation, Hangzhou Dianzi University, Hangzhou, China; 5 State Key Laboratory of Optoelectronic Materials and Technologies, School of Electronics and Information Technology, Sun Yat-Sen University, Guangzhou, China

## Abstract

**Background:**

Effective and accurate screening of oncological biomarkers in peripheral blood circulation plays an increasingly vital role in diagnosis and prognosis. High-sensitivity assays can effectively aid clinical decision-making and intervene in cancer in a localized status before they metastasize and become unmanageable. Meanwhile, it is equally pivotal to prevent overdiagnosis of non-life-threatening cancer by eliminating unnecessary treatment and repeated blood draws. Unfortunately, current clinical screening methodologies can hardly simultaneously attain sufficient sensitivity and specificity, especially under resource-restrained circumstances. To circumvent such limitations, particularly for cancer biomarkers from early-onset and recurrence, we aim to develop a universal plasmonic platform for clinical applications, which macroscopically amplifies multiplexed fluorescence signals in a broad spectral window and readily adapts to current assay setups without sophisticated accessories or expertise at low cost.

**Methods:**

The plasmonic substrate was chemically synthesized in situ at the solid–liquid interface by rationally screening a panel of reducing monosaccharides and tuning the redox reactions at various catalyst densities and precursor concentrations. The redox properties were studied by Benedict’s assay and electrochemistry. We systemically characterized the morphologies and optical properties of the engineered plasmonic Ag structures by scanning electron microscopy (SEM) and spectroscopy. The structure-fluorescence enhancement correlation was explicitly explained by the finite-difference time-domain (FDTD) simulation and a computational model for gap distribution. Next, we established an enhanced fluoroimmunoassay (eFIA) using a model biomarker for prostate cancer (PCa) and validated it in healthy and PCa cohorts. Prognosis was explored in patients subject to surgical and hormonal interventions following recommended PCa guidelines.

**Results:**

The monosaccharide-mediated redox reaction yielded a broad category of Ag structures, including sparsely dispersed nanoparticles (NPs) of various sizes, semi-continuous nanoislands, and crackless continuous films. Optimal broad-spectral fluorescence enhancement from green to far-red was observed for the inhomogeneous, irregularly-shaped semi-continuous Ag nanoisland substrate (AgNIS), synthesized from a well-balanced redox reaction at a stable rate mediated by mannose. In addition, different local electric field intensity distributions in response to various incident excitations were observed at the nanoscale, elucidating the need for irregular and inhomogeneous structures. AgNIS enabled a maximized 54.7-fold macroscopically amplified fluorescence and long-lasting photostability. Point-of-care availability was fulfilled using a customized smartphone prototype with well-paired optics. The eFIA effectively detected the PCa marker in cell lines, xenograft tumors, and patient sera. The plasmonic platform rendered a diagnostic sensitivity of 86.0% and a specificity of 94.7% and capably staged high-grade PCa that the clinical gold standard test failed to stratify. Patient prognosis of robotic-assisted surgeries and hormone therapies was non-invasively monitored following efficient medical interventions. The assay time was significantly curtailed on the plasmonic platform upon microwave irradiation.

**Conclusions:**

By investigating the effects of reducing monosaccharides on the seed-mediated chemical synthesis of plasmonic Ag structures, we deduced that potent multiplexed fluorescence enhancement originated from both an adequate reducing power and a steady reduction rate. Furthermore, the inhomogeneous structure with adequate medium gap distances afforded optimal multiwavelength fluorescence enhancement, thus empowering an effective eFIA for PCa. The clinically validated diagnostic and prognostic features, along with the low sample volume, point-of-care feasibility with a smartphone, and microwave-shortened assay time, warrant its potential clinical translation for widespread cancer biomarker analysis.

**Graphical Abstract:**

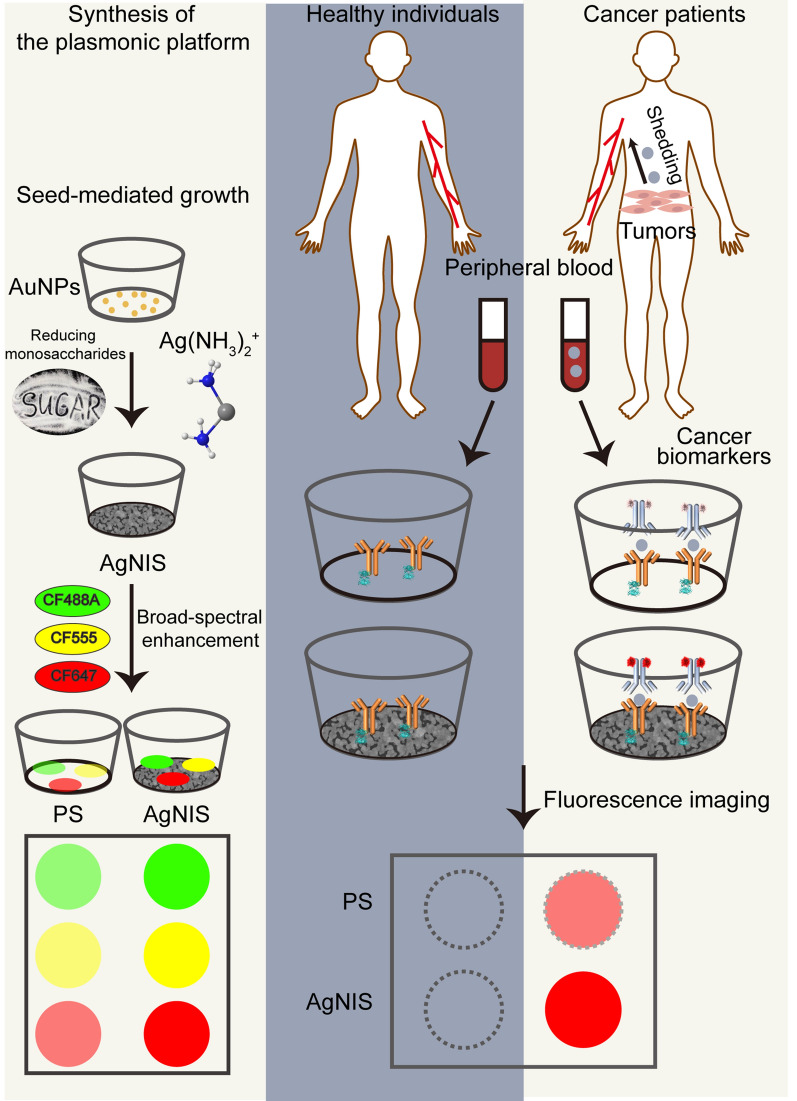

**Supplementary Information:**

The online version contains supplementary material available at 10.1186/s12951-022-01359-z.

## Introduction

Fluorescence-based quantitative assessment of the abundance of biomarkers has been entrenched among the most standard techniques in clinical cancer diagnosis. It is advantageous in superior sensitivity, extended signal stability, a broad dynamic range, and multiplexing capability. Nevertheless, the intrinsic low quantum yields and photobleaching susceptibilities of fluorophores severely hamper widespread applications where high accuracy and sensitivity are necessary. Therefore, extensive attempts have been made to amplify fluorescence signals, including synthesis of alternative fluorophores [1], optimization of chemical modification [2], engagement of complex secondary detection moieties such as up-conversion fluorescence NPs [3, 4], antibody-NP conjugates [5], and introduction of enzyme-catalyzed substrate [6]. The dielectric planar waveguide, as a sensitive alternative with improved fluorescence, is readily commercialized for protein analysis [7]. Another emerging strategy replies on the near-field optical phenomenon named metal-enhanced fluorescence (MEF), where well-matched resonant fluorophores positioned in precisely-controlled proximity to nanoscopic plasmonic surfaces are augmented, as a combined result of enhanced local excitation of electric fields, dipolar coupling of surface plasmons to fluorescence emission, and increased radiative decay rates of excited-state fluorophores [8, 9]. Since the introduction of the plasmonic resonance-controlled fluorescence [10, 11], significant advances have been attained to amplify fluorescence, using noble metal nanoarchitectures including but not limited to periodically ordered arrays [12–14], colloidal NPs in a variety of geometric shapes [15–18], and nanoislands on glass substrate [19, 20]. Given the warranted superiorities, MEF has been adopted with respect to clinical diagnosis for various ailments such as lung cancer [21], diabetes [22], cardiovascular diseases [23], and most recently, SARS-CoV-2 [24]. However, a vast majority of MEF platforms demand specialized, sophisticated, and laboratory-scale instruments that have not been widely available in clinical settings. Furthermore, the majority of contemporary studies on biomarker detection by MEF primarily focused on a modified glass substrate that usually requires a dust-free cleanroom for handling and manufacturing. Of utmost importance, it is convenient and inexpensive to retain existing in-clinic consumables and fluorescence equipment for signal readout.

Cancer biomarkers in the peripheral blood are known to shed from early-state cancers in living subjects, serving as valid reporters to monitor cancer progress and aggressiveness. In the context of the present study, we focused on PCa, which has been one of the most prevalent cancers in men, accounting for the leading cause of cancer death. In particular, non-metastatic PCa presents a nearly 100% 5-year survival rate which drastically drops to around 30% with metastasis, necessitating early diagnosis of malignancies [25]. The primarily prostate gland-localized kallikrein, prostate-specific antigen (PSA), has been extensively used as a biomarker for clinical management and screening, resulting in substantial mortality reduction partly due to declined metastatic incidences [26, 27]. Guidelines recommend PSA screening for the evaluation of men with high-risk factors. Although the diagnostic potency of PSA remains controversial due to relatively poor specificity and thus unnecessary overdiagnosis, the implication of accompanying PSA blood tests alongside digital rectal examination (DRE) and definitive biopsies have become clinically indispensable, as exemplified by Sangia and Elecsys total PSA tests that were already approved by the US Food and Drug Administration (FDA) [28]. In addition, ultrasensitive PSA assays are exceptionally beneficial for prognosis to identify recurrences after receiving curative procedures to guide timely salvage therapies [29]. Such assays with low detection limits allow sensitive surveillance of disease progresses ahead of clinical and radiological evidence, known as biochemical recurrence. Despite a few feasible finger-prick tests, the majority of prevalent tests, such as enzyme-linked immunosorbent assays (ELISA), usually require relatively high blood volumes, laboratory settings, and prolonged detection time. Accurate in vitro diagnostic strategies can significantly reduce healthcare expenses and adverse complications associated with invasive procedures of DRE and biopsies, such as irritation, discomfort, pain, infections, and hematuria. To that end, developing rapid, sensitive, and reliable diagnostic tools at the point of care would facilitate an optimal workflow of cancer management to direct accurate decision-making for clinicians, circumvent surplus overdiagnosis, and improve prognosis.

We, therefore, rationally screened a panel of common reducing monosaccharides and systemically interrogated their redox effects on the synthesis of Ag plasmonic structures on accustomed polystyrene (PS) substrate. Ag is a favorable metal species for multiplexed enhancement in the broad spectral range with strong surface plasmon and sharp angular resonance [30]. The redox reactions were kinetically tuned by catalyst densities and precursor concentrations. We addressed the structure-MEF correlation by FDTD simulation and established a gap distribution model for analysis. Structurally semi-continuous AgNIS, rather than its continuous or discontinuous counterparts, is advantageous with sufficient spectral overlaps between its extinction and multiple fluorochrome emissions that empower potent steady-state enhancement. We demonstrated an eFIA that facilitated simplified procedures, high sensitivity, stability, portability, and assay acceleration by microwave. It also correlated well with electrochemiluminescence immunoassays (ECLIA) in clinical practice. Besides the diagnostic value, prognostic use was validated in patients that received robotic-assisted laparoscopic radical prostatectomy (RALRP) or hormone therapies. AgNIS is thus a promising plasmonic platform that capably pinpoints diseased but probably asymptomatic states with non-invasiveness through intuitively detecting low-abundance cancer biomarkers beyond conventional assays in complex matrices.

## Materials and methods

### Materials

Gold chloride trihydrate (HAuCl_4_·3H_2_O), silver nitrate (AgNO_3_), cell culture-grade dimethyl sulfoxide (DMSO), and bovine serum albumin (BSA) were purchased from Sigma-Aldrich. All saccharides including d-glucose, d-mannose, d-xylose, d-galactose, d-maltose, sucrose, and l-arabinose were acquired from Shanghai Macklin Biochemical. Poly-l-lysine (PLL) and horseradish peroxidase-conjugated BSA (HRP-BSA) were obtained from Solarbio. Water-soluble amine-reactive N-hydroxysulfosuccinimidobiotin (sulfo-NHS-biotin) and streptavidin (SA) were bought from APExBIO. CF488A, CF555, and CF647 fluorescent dyes in NHS esters were attained from Biotium. Mouse anti-human PSA monoclonal antibody pairs (clones: 7H2 and 8A12 as capture and detection antibodies, respectively) and recombinant human PSA expressed in an *E. coli* system were purchased from GenScript. Phosphate-buffered saline (PBS) used in the study was 1 × dilution (10 mM Na_2_HPO_4_, 1.75 mM KH_2_PO_4_, 137 mM NaCl, and 2.65 mM KCl at pH 7.2–7.6) from a 20 × stock (Sangon Biotech) and autoclaved for sterilization before use unless otherwise noted. 18.2 MΩ-cm ultrapure Milli-Q® water (MilliporeSigma) was used in the entire study.

### Characterization

Ultraviolet–visible (UV–vis) absorption spectra were recorded using a UV-2600 spectrophotometer (Shimadzu) in a quartz cuvette or an Infinite M200 Pro multimode microplate reader (Tecan) in a microplate. All fluorescence measurements were conducted using the Tecan plate reader in ultrathin special optics PS microplates with minimal background fluorescence. Morphologies of nanostructures were examined by a JEOL JSM-6700F field emission-scanning electron microscope (FE-SEM) operating at 10 kV. Energy-dispersive X-ray spectroscopy (EDS) and elemental mapping were acquired with a high-resolution Zeiss Gemini500 thermal FE-SEM at 6 kV accelerating voltage. Surface hydrophobicity was indicated by water contact angles measured with a DSA100 Drop Shape Analysis System (Kruss) in ambient conditions. Protein secondary structures were assessed by a Nicolet 6700 Fourier transform infrared (FTIR) spectrometer (Thermo Fisher Scientific) in the attenuated total reflection (ATR) mode. All electrochemical measurements were performed on a CHI 630E Electrochemical Analyzer (CH Instruments) in a standard three-electrode configuration comprised of a glassy carbon working electrode, a Pt plate counter electrode, and a Ag/AgCl reference electrode.

### Synthesis of Ag nanoisland substrate

AgNIS was synthesized through a two-step surface-based seed-mediated growth, using a modified silver redox reaction with AuNPs as catalysts and nucleation centers [31]. First, AuNPs were synthesized using a modified Turkevich method [32, 33]. Succinctly, 15 mL 1% w/w sodium citrate was added to 100 mL 1 mM HAuCl_4_ for 15 min of boiling. After being cooled to room temperature (RT), the reaction mixture was passed through a 0.22 μm membrane filter. Subsequent synthesis of AgNIS was initiated by adding 50 μL preceding citrate-capped AuNPs solution as seeds to PS surface that was pretreated overnight at RT with 50 μL PLL in concentrations ranging from 0.001 to 1 mg mL^−1^. After 2 h of incubation at RT followed by washing, a freshly prepared Tollens’ reagent containing 24 mM NaOH, 13 mM NH_4_OH, and 20 mM AgNO_3_ to form the colorless Ag(NH_3_)_2_^+^ complex. 100 μL Tollens’ reagent at various concentrations was added to the AuNP-seeded surface, followed by 100 μL 10 mM reducing sugars of different kinds. The incubation was kept on a microplate shaker for 15 min for the growth of Ag nanostructures.

### Benedict’s assay

To correlate the reducing power of a variety of saccharides with Ag nanostructures and corresponding fluorescence enhancement, we conducted a semi-quantitative assay by preparing Benedict’s reagent constituted of 69 mM CuSO_4_, 0.95 M Na_2_CO_3_, and 0.67 M sodium citrate. 50 mM saccharide was mixed with the above Benedict’s reagent at a v/v ratio of 1:9 and incubated on a thermostatic water bath at 100 °C for 8 min. Absorption was measured at 260 nm, and the non-reducing disaccharide sucrose was used as a negative control.

### Determination of fluorescence enhancement

Native BSA was selected as the model molecular probe because its hydrodynamic radius of approximately 7 nm in ambient conditions provides a viable distance between fluorophores and plasmonic surface [34]. BSA was covalently modified with CF488A, CF555, and CF647 by amine-NHS crosslinking. Each fluorophore was dissolved in anhydrous DMSO and added to a pH 8 PBS solution containing 0.1 molar equivalence of BSA, with the final DMSO volume restricted below 0.5% v/v to advantageously reserve native protein structure. The reaction mixture was incubated on a shaker at RT for 2 h. Purification was performed with NAP size-exclusion chromatography columns (Cytiva). The resulting complexes were added to as-synthesized AgNIS for 2 h of incubation at RT and sealed from light until measurement of MEF at different wavelengths. Fluorescence intensities (FIs) were quantified in Quantity One software v4.6.6 (Bio-Rad) by measuring regions of interest (ROIs) of fluorescence images scanned with a Molecular Imager PharosFX Plus System (Bio-Rad) and a home-built microplate adaptor. Background (BKGD) was subtracted, and the enhancement factor was calculated according to the following equation:

$$EF \left( {Enhancement factor} \right) = \frac{{FI\left( {AgNIS} \right) - BKGD\left( {AgNIS} \right)}}{{FI\left( {PS} \right) - BKGD\left( {PS} \right)}}$$

### Finite-difference time-domain simulation

Optical simulations were performed with the commercial software FDTD Solutions (Lumerical). The structure of irregularly-shaped semi-continuous AgNIS was established by importing the corresponding SEM image with a selected region of size 2000 nm × 1500 nm on the PS substrate. The refined mesh area with a mesh size of 1 nm was utilized to cover the entire nanostructure to assure simulation accuracy. The dielectric function of Ag is based on the optical constants given by the CRC handbook [35].

### Determination of gap distance distribution

The distance distribution of inhomogeneous gaps was quantified by gap skeletonization and computation of gap distances. Firstly, we categorized all image pixels into binary gaps (0) or silver structures (1) through the *imbinarize* function in MATLAB software R2020b with global thresholding computed using Otsu's method [20], followed by skeletonization and distance computation. Next, the ‘clean’ operation of the *bwmorph* function was performed, followed by the ‘majority’ operation to optimize final binary images. The gap skeleton was acquired by inverting binary images with the *imcomplement* function and applying the 4-connectivity *bwskel* function to the inverted images to preserve the topology and Euler number of gaps. The gap distance is defined as twice the shortest distance from the medial skeleton line of gaps to the sliver structural boundary. Then, the Euclidean distance transformed by the* bwdist* function was applied to collect gap distances from each pixel to the nearest sliver boundary. Statistical analyses included only the Euclidean distance of the gap skeleton. Finally, the Distribution Fitter app in MATLAB was used for gamma distribution fitting.

### Synthesis of Ag nanoparticles

Ag nanoparticles (AgNPs) were synthesized using a solution-phase approach analogous to AgNIS. Briefly, 1 mL 100 mM mannose aqueous solution was added to 9 mL premixed AgNO_3_ and NH_4_OH at final concentrations of 1 mM and 5.9 mM, respectively. 10 mM NaOH was then introduced into the reaction to accelerate the reduction rate. The reaction mixture was maintained at RT for 10 min under stirring at 500 rpm. The morphology was visualized by a JEOL JEM-2010HR transmission emission microscope (TEM) operating at 200 kV, and the hydrodynamic size was measured by Zetasizer Nano ZSE (Malvern Panalytical).

### Preparation of continuous Ag thin films by magnetron sputtering

To investigate the influence of structural continuity on fluorescence enhancement, we synthesized a continuous Ag thin film on PS that was pretreated with 0.001 mg mL^−1^ PLL overnight at RT to increase substrate adhesion. The substrate was placed on the holder at a 20° angle of inclination to ensure successful deposition. Radiofrequency magnetron sputtering deposition was conducted with a silver target in magnetron discharge plasma at the sputtering power of 60 W for 5 min using a VTC-300 system (ZKDS Technology).

### Solution-phase synthesis of Au thin films

The PS surface pretreated with 0.001 mg mL^-1^ PLL was modified by AuNPs seeds identically as AgNIS. After washing three times with water to remove unbound AuNPs, it was immersed in a 10 mM equimolar solution of HAuCl_4_ precursor and hydroxylamine and placed on a shaker for 15 min at RT to complete the growth process.

### Cell lines and culture

Cell lines were all acquired from American Type Culture Collection (ATCC). PC-3 (derived from human prostate adenocarcinoma) and LNCaP (derived from human prostate carcinoma) were cultured in complete Gibco Dulbecco's Modified Eagle Medium (DMEM) media supplemented with 10% fetal bovine serum (Gibco) and 1% penicillin/streptomycin (Thermo Fisher Scientific). Cells were grown in a Heracell VIOS 160i incubator (Thermo Fisher Scientific) with a humidified atmosphere and 5% CO_2_ at a constant 37 °C. Cell counting was accomplished in PD100 counting chambers using a Cellometer Auto 1000 automated cell counter (Nexcelom Bioscience), following trypan blue staining.

### Western blotting

The 1 × cell lysis buffer was prepared by diluting a 10 × radioimmunoprecipitation assay (RIPA; 0.5 M Tris–HCl, pH 7.4, 1.5 M NaCl, 2.5% deoxycholic acid, 10% NP-40, 10 mM EDTA) buffer (MilliporeSigma) supplemented with phosphatase and protease inhibitor cocktails (Bimake). Cells were harvested with cell scrapers and lysed in cell lysis buffer on ice for 30 min. Lysates were cleared by centrifugation at 12,000 g for 20 min, with the supernatant transferred for normalization of concentrations using a BCA protein quantification kit (Pierce). Loading lysates were prepared by adding NuPAGE LDS sample buffer (Invitrogen) and reduced for 10 min at 100 °C. Proteins were separated by 10% sodium dodecyl sulfate–polyacrylamide gel electrophoresis (SDS–PAGE) and transferred onto methanol-activated Immobilon-P PVDF membranes (MilliporeSigma) at 210 mA constant current for 1 h. The blotted PVDF membranes were blocked with 3% BSA in 1 × TBST buffer (100 mM Tris–HCl, 150 mM NaCl, 0.5% Tween 20 at pH 7.5) at RT for 2 h. Membranes were then cut based on the molecular weight with reference to the protein ladder and incubated with the following primary antibodies (Proteintech) at 4 °C overnight: 1:5,000 mouse monoclonal anti-β-actin (clone: 7D2C10) and 1:2,000 rabbit polyclonal anti-PSA. After being washed with 1xTBST on a shaker for 30 min, 1:10,000 corresponding HRP-conjugated goat anti-mouse or anti-rabbit IgG secondary antibodies (Proteintech) were added for a 1 h incubation at RT. Membranes were scanned with a Bio-Rad ChemiDoc™ Touch Imaging System with a chemiluminescence kit (Bio-Rad).

### Multicolor imaging using an integrated smartphone prototype

A field-portable fluorescence imaging setup was enabled by a smartphone. The box module was digitally designed in SolidWorks software and printed via stereolithography with photosensitive curing resins by a UnionTech Lite 300 3D printer. The BP filter adaptors were cuboids with a length of 200 mm and a width of 33 mm to accommodate filters of all sizes. Two sets of band-pass (BP) filters (Shenzhen Yu Sheng Electronic Technology) consisted of 470 ± 10, 550 ± 10, 650 ± 10 nm for excitation, and 520 ± 10, 590 ± 10, and 680 ± 10 nm for emission, respectively. The filters were placed into 3D-printed inserts that situated the module. A 10 W integrated white-light LED was utilized to introduce BP-filtered illumination as the excitation source and set apart from the imaging window to create an incident oblique excitation and minimize excitation interference. The imaging window was set as a square with a side length of 15 mm. The power supply of LED was transformed by a 220 V AC to 12 V DC inverter to prevent undesirable stroboscopic effects. The height of the module was adjusted to 157 mm for observable focus planes on well plates. The length and width of the module were adjusted to 167 and 127 mm, respectively, to support most smartphones available in the market. BP interference filters for emission were installed in front of the camera to reject scattered and reflected excitation light and optimize fluorophore-specific emission.

### Biotinylation of BSA

BSA was biotinylated at the reactive lysine side chains by adding 30-fold molar equivalence of sulfo-biotin-NHS to a BSA solution in pH 8 PBS. After 2 h incubation at RT, excessive biotin was removed by NAP purification columns. Next, the biotin labeling efficiency was quantified using 4'-hydroxyazobenzene-2-carboxylic acid (HABA). HABA binds SA to produce a colorimetric complex with strong absorption at 500 nm. Solvent-accessible biotin on BSA displaces bound HABA for its higher affinity with SA, causing absorption to decrease proportionately. The HABA-SA mixture of 0.5 mg mL^−1^ SA and 300 μM HABA was prepared in PBS. A fivefold dilution of biotin-BSA was added to the HABA-SA mixture and equilibrated for 30 s to record changes in OD_500_.

### Covalent modification of capture antibody with SA

Primary amine groups of the anti-PSA capture antibody (cAb) were modified into sulfhydryl groups using Traut’s reagent (2-iminothiolane) at a molar ratio of 1:10 in pH 8 PBS (5 mM EDTA, DMSO < 1% v/v) for 30 min. The thiolated cAb was then purified by ultracentrifugation. In the meantime, amines of SA were modified into maleimides by sulfosuccinimidyl 4-(N-maleimidomethyl)cyclohexane-1-carboxylate (sulfo-SMCC) in pH 7.4 PBS at a molar ratio of 1:10. After 1 h of incubation, the activated SA was purified by ultracentrifugation and then mixed with the thiolated cAb in pH 7.4 PBS containing 5 mM EDTA in threefold molar excess. Finally, the reaction was rotated at RT for 1 h, and the SA-cAb complex was purified by an Amicon® MWCO 100 kDa ultracentrifuge filter (MilliporeSigma).

### Enhanced fluorescence immunoassay on AgNIS

PS or AgNIS was immersed in 20 μg mL^−1^ biotin-BSA in PBS at 4 °C overnight. Wells were then washed three times with PBS and blocked with 3% BSA in PBS for 30 min, followed by 1 h incubation at RT in a 0.1% BSA PBS solution with 15 μg mL^−1^ SA-cAb. After washing three times with PBS, 50 μL PSA calibration standards at various concentrations in 0.1% BSA PBS were added for a 1 h duration of antibody-antigen interaction at RT. Immediately after washing, wells were incubated in 50 μL 0.1% BSA PBS containing 5 μg mL^−1^ CF647-labeled anti-PSA detection antibody (CF647-dAb) for 1 h at RT. At last, they were washed, dried, and scanned for fluorescence. The putative assay time starting from antigen addition was approximately 2 h. 10 μL samples were diluted to 50 μL with 0.1% BSA in PBS for serum measurement. Limit of detection (LOD) was calculated as 3 times of standard deviation (s.d.) of the blank with no antigens.

### Conventional ELISA

High-bind 96-well PS microplates (Corning) were used for ELISA with the same antibody pair. The plate was coated with 50 µL 5 µg mL^−1^ cAb at 4 °C overnight, followed by blocking with 3% BSA in PBS for 1 h at RT. Identical PSA standards as in eFIA were applied for 1 h incubation at RT. Following plate washing, 2 µg mL^−1^ biotin-dAb in 0.1% BSA PBS was dispensed and incubated for 1 h. Subsequently, the plate was washed and incubated in a 1:2,000 dilution of SA-HRP (Beyotime) for 1 h. After washes, 100 µL 3,3′,5,5′-tetramethylbenzidine (TMB, Beyotime) substrate was added to each well for 30 min with reaction stopped by a half-volume 2 M H_2_SO_4_. Optical density (OD) was measured at 450 nm for quantification.

### Detection of secreted PSA in PCa cell culture with AgNIS

The expression and shedding of PSA in PC-3 and LNCaP human PCa cell lines were studied by eFIA on AgNIS. First, 1 × 10^6^ cells per well were seeded in a 6-well plate and cultured for 12 h in a 37 °C incubator. The culture media were then replaced with serum-free DMEM media containing DMSO, dihydrotestosterone (DHT, TargetMol), enzalutamide (ENZA, Shanghai Aladdin Biochemical Technology), or DHT plus ENZA (DMSO v/v < 0.5% in all cases to avoid cytotoxicity) for another 24 h incubation. At last, cells were harvested and lysed for expression analysis. Meanwhile, the culture media were also collected and centrifuged at 5,000 rpm for 10 min to remove any cell fragments, with supernatants subject to secretion analysis.

### Detection of PSA in PCa tumors of human xenograft models

Inbred homozygous (Foxn1^nu^/Foxn1^nu^) mutant male BALB/c nude mice of 4–6 weeks were purchased from GemPharmatech and used for tumor inoculation in agreement with the animal protocol #L102012019120A approved by the Institutional Animal Care and Use Committees at Sun Yat-sen University Cancer Center (SYSUCC). Typically, 3–10 × 10^6^ PCa cells in a 200 μL media:Matrigel (Corning) mixture (2:1 v/v) were injected subcutaneously near the right upper-flank. Xenograft mice bearing PC-3 and LNCaP tumors (n = 3) were sacrificed 30 and 20 days after grafting, respectively. Subcutaneous tumors were resected and placed in an Eppendorf tube containing 1 × RIPA lysis buffer with protease inhibitors and two ceramic grinding beads. Tissues were homogenized for 10 min at 70 Hz and -35 °C with a Luka Bead-mill Tissue Lyser (Guangzhou Luka Sequencing Instruments). Lysates were centrifuged at 12,000 g for 20 min with supernatant collected for analysis. Prostate glands (n = 3) were included as controls.

### Microwave acceleration

Microwave acceleration was experimentalized on the interaction between AgNIS surface-immobilized biotin-BSA and CF647-labeled streptavidin (SA-CF647) under microwave irradiation. The microwave power and irradiation time were precisely controlled by an MCR-3E Lab Chemistry Microwave Reactor (Zhengzhou Yarong Instrument). The unmodified AgNIS surface without biotin-BSA was irradiated as a control for background subtraction. To best preserve native antigenicity of PSA in initial eFIA steps, the microwave was only applied to facilitate the binding of captured PSA and CF647-dAb. The instantaneous temperature elevation on the plasmonic surface in response to the microwave was measured in PBS with a FLIR ONE Pro thermal imaging camera.

### Human serum

Approval for the human protocol #B2019-121-01 in this study was acquired from the SYSUCC ethics committee for investigational purposes. Sera from a cohort comprised of PCa patients (n = 50) and healthy subjects (n = 19) were collected on-site or from the Tumor Biobank at SYSUCC between 2019 May and 2020 Nov. All PCa patient samples were confirmed upon diagnosis by histopathological examination. Pre-interventional blood samples were drawn in venipuncture tubes on the day of interventions. In contrast, post-interventional blood was collected 4 days after RALRP or hormone therapy based on the TNM staging. After being centrifuged at 2,000 rpm for 10 min, serum and plasma were preserved from gel yellow-topped (clot activator and serum gel separator) and green-topped (sodium heparin) venipuncture tubes, aliquoted, and stored at -80 °C before use. Anticoagulated whole blood was kept at 4 °C and tested within 7 days following collection. Clinical PSA levels were measured with an Elecsys® total PSA kit on the Roche cobas e 602 analyzer by ECLIA.

### Statistical analysis

Statistical analysis was performed using Microsoft Excel and SPSS Statistics. Data were presented as mean ± s.d. denoted by error bars from a minimum of three replicates unless otherwise noted. Statistical significance was assessed by two-tailed paired or unpaired Student’s *t*-tests. Neither blinding nor randomization was performed on the subjects. No prior statistical analysis was used to predetermine the sample size for enrollment.

## Results and discussion

**Fig. 1:**
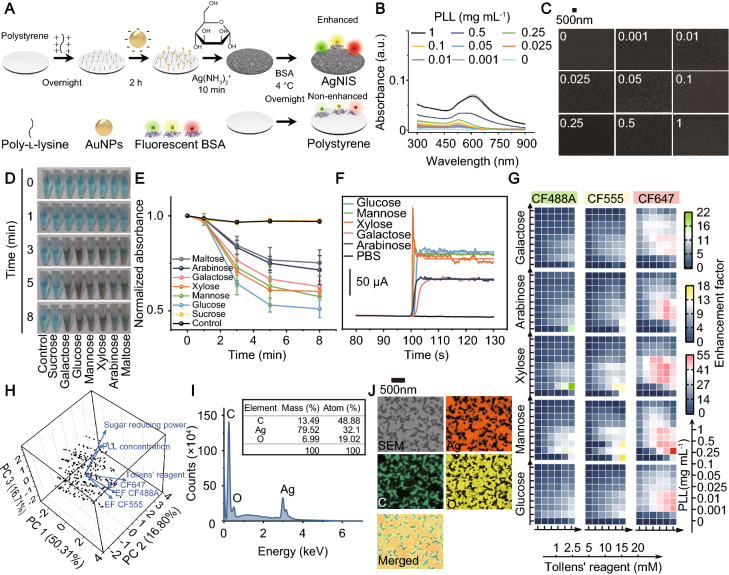
Chemical synthesis and characterization of AgNIS for MEF. **A** Schematic illustration of the AuNP-catalyzed synthesis of AgNIS with broad spectral fluorescence enhancement. **B** UV–vis absorption spectra and **C** representative SEM images of AuNP-immobilized PS substrate at different seeding densities mediated by PLL concentrations. Shaded boundaries represent confidence intervals (CI) within ± 1 SD from three independent samples. **D** Photographs and **E** normalized absorption at 260 nm of the time-course Benedict’s assay for 50 mM reducing monosaccharides (n = 2). Solutions with no saccharides and the non-reducing disaccharide sucrose were introduced as controls. **F** Amperometric i-t curves of 10 mM saccharides in 0.1 M pH 7 PBS at a constant potential of + 1.5 V with a glassy carbon working electrode. **G** Heatmap showing fluorescence enhancement of fluorophore-labeled BSA probes on Ag structures. The Ag synthesis advanced at equivalent concentrations of reducing monosaccharides and varied in gradient concentrations of PLL and Tollens’ reagents that controlled seeding densities and growth availabilities. **H** 3D PCA biplot of Ag structures synthesized under different conditions. 83.82% of the total variance is explanatory by the first three components, with eigenvalues above the retention cut-off conforming to the Kaiser-Guttman rule (Additional file 1: Fig. S10). The loading vectors of PLL, Tollens’ reagent, and sugar reducing power diverge from those of EFs, which form small angles by themselves. **I** The energy-dispersive X-ray spectrum (EDS) of AgNIS. **J** Elemental mapping analysis on the nanostructural AgNIS. Nanoisland-like structures are primarily elemental silver, with low levels of oxygen intensities. Carbon originates from the PS substrate

The overall synthesis started with ionic surface modification by PLL (Fig. 1A), which allowed strong electrostatic interactions with negatively charged AuNPs. As a result, AuNP seeds were efficiently immobilized on the surface. After the subsequent interaction between the positive-valent Ag(NH_3_)_2_^+^ complex and AuNPs, the in situ redox reaction proceeded in alkaline with monosaccharides that underwent intramolecular ring-open reactions to form reducing cyclic hemiacetals. Metallic Ag was deposited around AuNPs nucleation centers, followed by further expansion via autocatalytic growth. The density of AuNPs as nucleation sites increased with ionic PLL concentrations, prompting an aggregation-associated bathochromic peak shift in UV–vis absorption (Figs. 1B and Additional file 1: Fig. S1). SEM also visualized the tendency, with clustered and agglomerated AuNPs observed at high PLL concentrations (Fig. 1C). Next, we examined a number of monosaccharides with hemiacetals as reducing agents for Ag reduction by Benedict’s assay (Figs. 1D and Additional file 1: Fig. S2A, B). While the non-reducing sucrose did not induce reduction, glucose and maltose presented the most potent and weakest reduction, respectively (Figs. 1E and Additional file 1: Fig. S2C). In contrast, mannose demonstrated a well-balanced reducing power with a more stable rate than glucose and xylose (Additional file 1: Fig. S2D). It was in agreement with the observation from cyclic voltammetry (Additional file 1: Fig. S3) and amperometry (Fig. 1F), where glucose, mannose, and xylose displayed potent oxidation current. The monosaccharides were then used as the reducing agent at various synthesis conditions, each engendering metallic Ag structures of different plasmonic properties (Additional file 1: Figs. S4–S8).

Proteins like albumins are exhaustively documented to have high binding affinities to metals and form corona layers [32, 36], which are an ideal model probe to provide an appropriate distance for enhancement and prevent fluorophores in close proximity to the plasmonic surface from quenching. Using fluorophore-labeled BSA, we scrutinized the macroscopic enhancement on a broad spectral range of fluorescence across the visible window (Additional file 1: Fig. S9). Xylose and mannose, in general, exhibited higher EFs than the most reducing glucose and other less reducing monosaccharides (Fig. 1G), owing to their well-balanced reducing power and kinetics. Robust reduction by glucose was not optimal because of unstable reaction kinetics and fast reaction rates, which are unfavorable to control the diffusion-based reaction and maintain balanced local reactant concentrations at the solution-surface interface (Additional file 1: Fig. S2D). This conclusion was reinforced by the principal component analysis (PCA). The fluorescence enhancement of all colors was strongly associated with each other and only marginally correlated to Ag precursors but irrelevant to PLL or reducing monosaccharides (Figs. 1H and Additional file 1: Fig. S10). The optimal enhancement was observed to be 21.9- (CF488A; xylose), 17.6- (CF555; mannose), and 54.7-fold (CF647; mannose) (Fig. 1G). Due to the potent fluorescence enhancement in all colors identified in the screening, mannose was selected for the balanced reduction necessitated for MEF.

To systemically elucidate the structure-MEF correlation for mannose-mediated synthesis, we conducted extensive morphological investigations by SEM on Ag structures under a diverse range of synthesis conditions, entailing different seeding densities and precursor concentrations (Fig. S11). Quantified by an automated thresholding approach, the Ag surface coverage gradually increased with elevated concentrations of PLL and Ag precursors (Additional file 1: Fig. S12), while discrete small nanoparticle structures progressively coalesced into intermittently gapped and eventually uninterrupted continuous structures (Additional file 1: Fig. S11). It is noteworthy that only homogeneously dispersed small nanoscopic particles formed without catalysis of AuNPs, at a low reaction efficiency leading to much lower Ag coverages in all conditions. Among the comprehensive structures screened, the optimal structure concurrently with the highest EF of CF647 and high EFs of CF488A and CF555 was tortuous irregular nanoislands in semi-continuity with a medium Ag coverage and gaps in different widths. The spatially non-uniform plasmonic structures can increase electric fields and confine light propagation at multiple wavelengths within the inhomogeneous microenvironment. In addition, the inter-gap space can well accommodate fluorescence molecules for localized enhancement. Characterization by elemental mapping and EDS evidenced the presence of Ag (Figs. 1I, J). The height of AgNIS was measured to be 116.4 ± 20.5 nm (Additional file 1: Fig. S13).

**Fig. 2:**
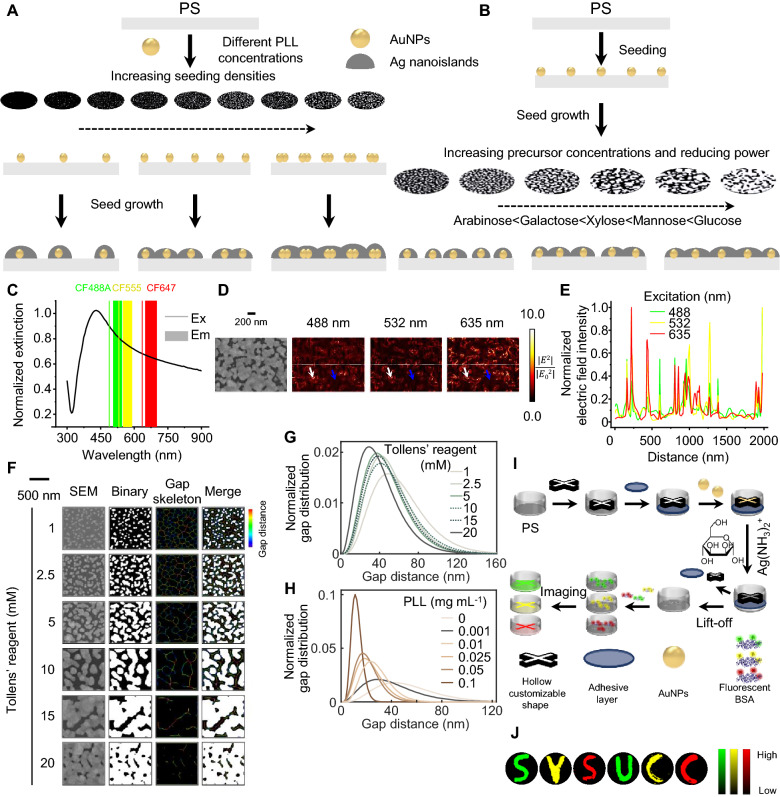
Mechanisms for precisely-controlled synthesis and broad spectral MEF of AgNIS. Schematic illustration for proposed mechanisms of Ag structures synthesized at **A** different seeding densities of AuNP catalysts and **B** different Ag precursor concentrations and reducing monosaccharides. In general, higher seeding densities, precursor concentrations, and reducing monosaccharides lead to higher degrees of structural continuity. **C** Extinction spectrum of AgNIS overlaid with excitation (lines) and emission (areas) of CF488A, CF555, and CF647 across a broad visible spectrum. **D** Electric field intensity distribution contours of AgNIS irradiated with polarized incident excitations at 488, 532, and 635 nm. White and blue arrows indicate gap and surface enhancements, respectively. **E** Line profiles of electric field intensities at different wavelengths indicated by the dotted lines in Fig. 2D. **F** Quantification of inhomogeneous gap distances for silver structures synthesized at a fixed 0.001 mg mL^−1^ of PLL and various Tollens’ reagent concentrations. Automated thresholding on SEM images was first executed to acquire binary images with silver and gaps depicted in white and black, respectively. The gap distance is defined as twice the shortest distance from the 4-connectivity skeleton of gaps to the sliver structural boundaries and is shown in the color scale. Normalized gap distributions of Ag structures synthesized at different concentrations of **G** Tollens’ reagents (PLL: 0.001 mg mL^−1^) and **H** PLL (Tollens’ reagent: 20 mM), fitted by the gamma distribution function on histograms (Additional file 1: Fig. S16). **I** Schematic diagram of the lift-off micropatterning procedures for controlled synthesis of AgNIS patterns. **J** Plasmon-enhanced multicolor fluorescence on micropatterned AgNIS of exemplified designed letters

The mechanism of optimal synthesis for the inhomogeneous semi-continuous island-like structures can be attributed to two major factors: (1) the seeding density of AuNP catalysts (Fig. 2A) and (2) the catalyzed silver reduction reaction jointly determined by the silver precursor concentration and a well-balanced reducing power (Fig. 2B). The unique asymmetric morphogenesis with optimal enhancement occurs without a preformed template at the rigid condition of a low seeding density and a high growth concentration. Consequently, it bears plasmon resonances well-overlaid with multiple fluorophores across the visible spectrum (Fig. 2C). While fluorescence of the short-wavelength BV421 residing in non-resonant regions was considerably quenched, appreciable MEF was observed for all green, yellow, and far-red fluorophores (Additional file 1: Fig. S14). In addition, AgNIS, after long-term storage in a vacuum, could stably sustain the fluorescence with improved photostability (Additional file 1: Fig. S15). To more comprehensively study MEF at the microscopic scale, we performed FDTD simulations at excitations of multiple wavelengths based on the morphological structures (Fig. 2D). The majority of electric field intensities were attributed to the edges of gap junctions, whereas a small proportion of weaker effects were found at antenna-like protrusions. The line profiles clearly showed different electric field intensity distributions in response to various incident excitations (Fig. 2E), clarifying the necessity of irregular and inhomogeneous structural configurations for fluorescence enhancement in a broad spectrum. To elaborate upon the gap distance distribution, we applied skeletonization and computation to acquire Euclidean distances in a line segment [20] (Figs. 2F and Additional file 1: Fig. S16L). Gaps of discontinuous and continuous structures are in a more significant number and a narrower width, respectively. Therefore, an effective gap distance as in AgNIS should be abundant while simultaneously large enough to accommodate gap-situated BSA molecules and small enough with potent MEF (Figs. 2G-H and Additional file 1: Fig. S16).

In contrast to AgNIS, mannose-reduced colloidal AgNPs of 55.8 ± 7.4 nm in size induced quenching for CF488 and minor enhancement for CF555 and CF647 (Additional file 1: Fig. S17), similar to Cy3 and Cy5 observed for citrate-reduced AgNPs [37]. The magnetron-sputtered Ag film weakly enhanced fluorescence (Additional file 1: Fig. S18), whereas the Au film autonomously synthesized similarly by AuNP seeds experienced quenching at shorter wavelengths due to structural continuity and unmatched surface plasmon (Additional file 1: Fig. S19). In the light of broad-spectrum MEF, the asymmetric island-like nanostructure of AgNIS is undoubtedly indispensable, which can be deemed an intermediate between AgNPs and continuous Ag films. Next, we developed a facile lift-off micropatterning approach for the selective and controlled synthesis of practically any predefined patterned shapes (Fig. 2I). The multicolor fluorescence intensities in letters were consistently higher than the surrounding PS surface without MEF (Fig. 2J).

**Fig. 3:**
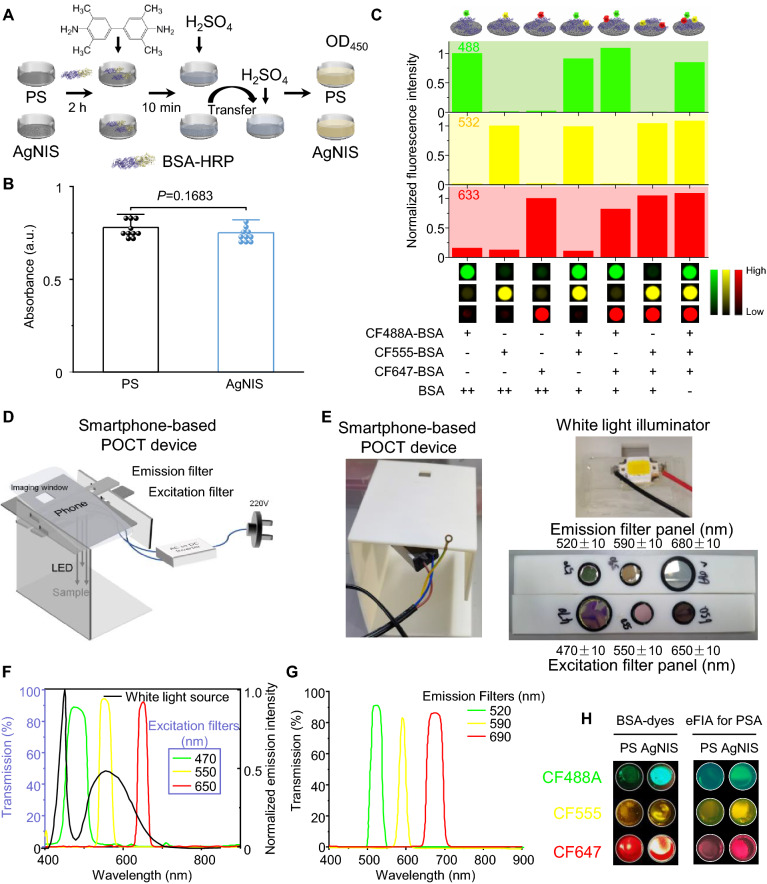
Fluorescence multiplexing and point-of-care capabilities of AgNIS. **A** Estimation of surface-immobilized BSA by a non-fluorescence enzymatic assay using BSA-HRP complexes. Assay solutions after enzymatic reactions were transferred into clear-bottomed wells for more accurate measurement of OD at 450 nm to circumvent intrinsic absorption interference from AgNIS. **B** Absorbance at 450 nm from BSA-HRP adsorbed on PS and AgNIS after enzymatic reactions. No statistical significance (*P* = 0.1683) was found (n = 10). **C** Multiplexed fluorescence verification of CF488A, CF555, and CF647 on AgNIS. Native BSA was included for concentration compensation. Specific fluorescence can be simultaneously and accurately differentiated in the corresponding channel. **D** Schematic design and **E** photographs of a smartphone-based POCT prototype for multiplexed fluorescence imaging. White light LED inverted to DC was used for illumination with appropriate excitation filter sets. **F** Transmission spectra of the excitation filters overlaid with the emission spectrum of the white-light excitation source (black). **G** Transmission spectra of the emission filters set in front of the camera for fluorescence detection. **H** Representative fluorescence images were scanned with the POCT prototype for PS and AgNIS modified with fluorophore-labeled BSA (left panel) or with immunoassay detection of 100 nM PSA (right panel)

It is then interrogated whether the fluorescence enhancement of AgNIS was due to broad spectral surface plasmon resonance, rather than merely more surface-absorbed molecules, on the grounds that AgNIS could have a slightly larger effective surface area as well as notable protein-nanostructure interactions [32]. Surface hydrophobicity was determined upfront by measuring the water contact angle, unchanged for AgNIS (Additional file 1: Fig. S20). Next, a non-fluorescent analog BSA-HRP was validated to adsorb at similar levels on both PS and AgNIS through enzymatic assays (Figs. 3A-B), indicative of the comparable macroscopic surface area. We then explored the multiplexing capability of AgNIS using mixtures of multicolor fluorescent probes that were excited at their respective wavelengths. Single, dual, and triple colors were effectively diverged and differentiated with minimal bleed-through noise and spectral interference, indicating the potential for multiplexed enhanced fluorescence detection (Fig. 3C). To achieve point-of-care testing (POCT), especially in remote and resource-limited settings without the need for sophisticated devices, we fabricated a prototype module by 3D printing which was adapted to a standard consumer cell phone. A white-light LED along with an appropriate excitation filter set, a panel of BP emission filters facing the camera, and an AC-to-DC inverter was integrated for qualitative imaging (Figs. 3D, E). All filters were validated for high transmission efficiencies > 80% and narrow bands by spectroscopy (Figs. 3F-G). In this preliminary prototype, the fluorescence of all colors was imaged to be markedly enhanced on AgNIS for eFIA (Fig. 3H).

**Fig. 4:**
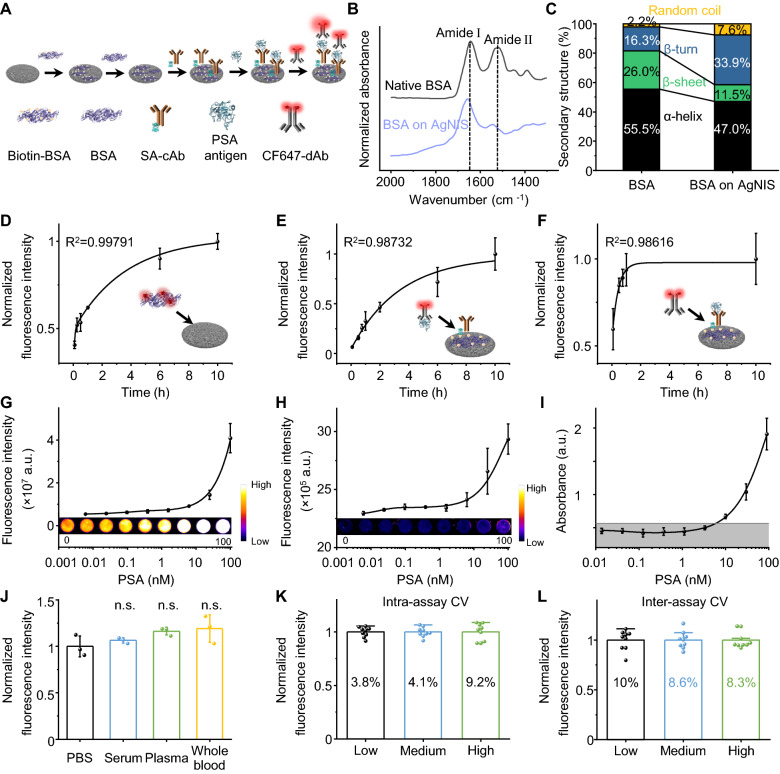
Enhanced fluorescence immunoassays on plasmonic AgNIS for highly sensitive detection of PSA. **A** Schematic of the sandwich immunoassay format implemented with biotin-BSA. CF647 fluorescence label with optimal enhancement on AgNIS was selected for signal readout. **B** ATR-FTIR spectra of native BSA and BSA on AgNIS. Dotted lines indicate typical amide I and II bands. **C** Conformational changes of BSA in the secondary structure after immobilization on AgNIS. Kinetic curves for dynamic binding processes of **D** BSA and AgNIS, **E** cAb and antigen, **F** antigen and dAb using CF647-BSA, CF647-dAb-antigen, and CF647-dAb probes, respectively. Calibration curves of the fluorescence signal intensity as a function of PSA concentrations for **G** AgNIS and **H** PS. Inset shows the corresponding fluorescence images. **I** Calibration curve of sandwich-type conventional ELISA for PSA with HRP-labeled dAb. Absorbance was measured at 450 nm with TMB substrate. **J** Comparison of signal intensities for 30 nM PSA spiked in PBS, serum, plasma, and whole blood on the AgNIS platform. Data were normalized by the mean fluorescence intensity (MFI) of PBS. One-way analysis of variance (ANOVA) Tukey's HSD post hoc test was used for statistical analysis. Evaluation for **K** intra-assay and **L** inter-assay coefficients of variability (%CV) at low (0.3 nM), medium (3 nM), and high concentrations (30 nM) of PSA

**Fig. 5:**
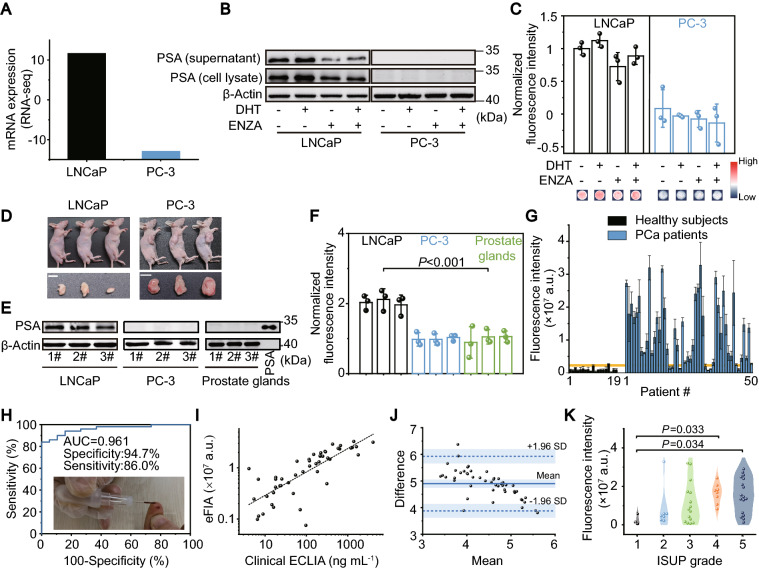
Detection of PSA in PCa cell lines, xenograft tumors, and human serum by AgNIS. **A** The mRNA expressions of PSA in PCa cell lines of LNCaP and PC-3 as retrieved from the Cancer Cell Line Encyclopedia (CCLE). **B** Western blots of cellular (cell lysate) and shedded (supernatant) PSA in LNCaP and PC-3, respectively, with high and low expressions. The expression and secretion of PSA in LNCaP were effectively induced and inhibited by DHT and ENZA, respectively, while no pharmacological effects were observed in PC-3. β-Actin was included as the internal loading control. **C** Detection of PSA in LNCaP and PC-3 cells using AgNIS upon treatment of DHT and ENZA. **D** Photographs of LNCaP and PC-3 human PCa xenograft mouse models and dissected tumors. Scale bars: 0.5 mm. Detection of PSA in PCa tumors from LNCaP and PC-3 xenografts by **E** immunoblotting and **F** eFIA on AgNIS. Autologous mouse prostate glands (without cross-species affinities) and PSA were included as negative and positive controls, respectively. **G** Serum eFIA tests of PSA in cohorts of PCa patients (n = 50) and healthy subjects (n = 19). The yellow line indicated the cut-off MFI value calculated from the ROC curve with the optimal sensitivity and specificity. **H** ROC curve of eFIA for PCa diagnosis. Inset: an example photograph showing the low blood/serum volume required for eFIA. **I** Scatter plot showing a good linear correlation between eFIA and clinical ECLIA tests (R = 0.782). **J** The Bland–Altman plot comparing eFIA and clinical ECLIA. 95% confidence intervals for LOA are defined by the mean ± 1.96 s.d. of differences. **K** Violin plots showing the correlation of serum PSA measured by eFIA with ISUP grades. Statistical analysis was performed by one-way ANOVA with Fisher's Least Significant Difference (LSD) Test

Given the excelling MEF, we proposed an eFIA to detect total PSA with AgNIS (Fig. 4A). The degree of labeling (DoL) in biotin-BSA was quantified 7.11 by the HABA assay (Additional file 1: Fig. S21A). The presumably labeled Lys residues as the most solvent-accessible are evenly located on the surface, making them sterically available for side-chain reactions (Additional file 1: Fig. S21B-D). Such biotinylation minimally influenced BSA structures (Additional file 1: Fig. S22A), and the modified biotin-BSA could be efficiently adsorbed on the AgNIS surface (Additional file 1: Fig. S22B). Upon direct adsorption on AgNIS, the conformationally sensitive amide II band of in-plane N–H bending and C-N stretching vibrations as probed by ATR-FTIR were greatly interfered with an apparent decrease [38] (Fig. 4B), demonstrating the strong interaction between BSA and AgNIS. Peaks in the Amide I region were deconvoluted by multipeak fitting to derive conformational variations of secondary structures (Additional file 1: Fig. S22C-D). Fractions of rigid structures like α-helices and β-sheets were observed to decrease, similar to the observation at the AuNP-protein interface [32], with only a minor increase in random coils (Fig. 4C). The increase in β-turn content could originate from the formation of intermolecular hydrogen bonds because of locally aggregated domains from partially regional unfolding [39]. After immobilizing SA-cAb through interactions with biotin-BSA, the eFIA proceeded in a standard sandwich ELISA format with a CF-647 labeled dAb. We systemically monitored the kinetics of eFIA. The pre-assay BSA immobilization process was relatively slow, with 10 h to reach saturation (Fig. 4D). The dynamic recognition of antigen by cAb was tracked by a fluorescent antigen-CF647-dAb complex (Fig. 4E), which turned out to be the rate-limiting step of eFIA as opposed to 1 h of rapid saturation for dAb recognition (Fig. 4F). Fluorescence on AgNIS increased with increasing concentrations of PSA in an exponential fit (R^2^ = 0.999) and was significantly higher than that of PS (R^2^ = 0.982) (Figs. 4G-H). The limit of detection (LOD) for eFIA was calculated to be 66 pM at a signal-to-noise ratio of 3, which is lower than recently published studies [40–43] (Additional file 1: Table S2) and almost two orders of magnitude lower than that of PS (3.0 nM) or a conventional HRP-based ELISA (5.9 nM) (Fig. 4I). The eFIA platform worked rigorously towards PSA in PBS, whole blood, processed serum, and plasma (Fig. 4J). Lot-to-lot reproducibility of AgNIS was confirmed by the intra- and inter-assay coefficients of variability (CV) against 0.3, 3, and 30 nM PSA, with all outcomes < 10%, proving its reliable technical accuracy (Fig. 4K, L). To determine intracellularly expressed and secreted PSA with AgNIS, we selected PCa cells of LNCaP and PC-3 corresponding to high and low PSA mRNA expressions, respectively (Fig. 5A), consistent with their protein expressions and antigen shedding by immunoblotting (Fig. 5B). In addition, the androgen hormone DHT, which is involved in the pathogenesis of benign prostatic hypertrophy and clinically prescribed for male hypogonadism, could promote the expression and secretion of PSA in LNCaP but exert no influence on PC-3, whose baseline expression was low (Fig. 5B). By contrast, ENZA, as an FDA-approved drug for metastatic castration-resistant PCa, can inhibit such a potent androgenic effect, even in the presence of the DHT inducer (Fig. 5B). These in vitro pharmacological effects could be saliently identified by AgNIS in congruency (Fig. 5C). Similarly, the expression of PSA in LNCaP tumors from xenograft mice was considerably higher than in PC-3 tumors (Figs. 5D-E), which was precisely detected by eFIA likewise (Fig. 5F).

Besides preclinical usefulness, we further demonstrated the clinical diagnostic values of AgNIS for PCa. 10 μL serum samples from cohorts of 50 PCa patients and 19 healthy subjects with matched age and male gender (*P* > 0.05) were collected and analyzed by eFIA (Additional file 1: Table S1). In general, the fluorescence intensities of PCa patients were much higher than those of healthy controls (Fig. 5G), apparently stratifying the two cohorts with different disease statuses by elevated serum PSA levels. It afforded a sensitivity of 86.0% and a specificity of 94.7% with an area under the curve (AUC) of 0.961 by the binary receiver operating characteristic (ROC) curve (Fig. 5H). The diagnostic performance of AgNIS is superior over the test characteristics of the most widely accepted balanced cutoff at 4.0 ng mL^−1^, with a sensitivity of 21% (51% for high-grade PCa) and a specificity of 91% [44]. In the meantime, the small serum volume can preclude venipuncture procedures in the clinic and potentially substitute it with finger pricking. We then used a quantitative total PSA ECLIA on the Roche clinical platform in parallel with eFIA, resulting in an acceptable correlation coefficient of 0.782 (Fig. 5I). The discrepancies between the two assays were assessed by the Bland–Altman analysis within the 95% limits of agreement (LOA), reaching a satisfactory agreement (Fig. 5J). Furthermore, we pathologically evaluated the Gleason Scores of biopsies and stratified the PCa patients according to the International Society of Urological Pathology (ISUP) grading system. Notably, ISUP Grade Groups 4 and 5 at higher risks with Gleason Scores > 8 can be accurately differentiated from the indolent ISUP Grade Group 1 (Fig. 5K), which ECLIA failed to identify (Additional file 1: Fig. S23).

**Fig. 6:**
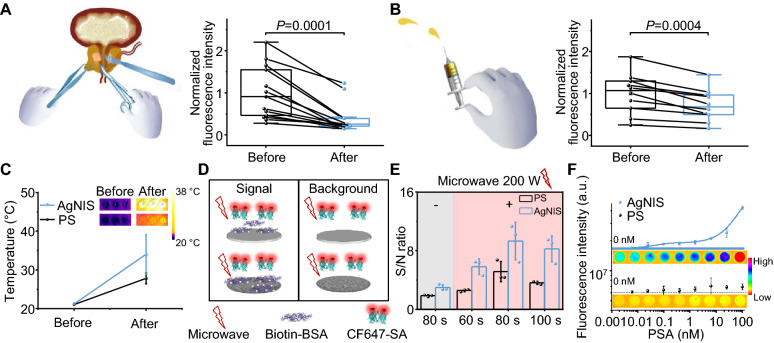
Clinical prognosis of PCa and microwave-accelerated eFIA with AgNIS. Serum eFIA tests to monitor **A** RALRP in non-metastatic PCa patients (n = 13), and **B** hormone therapy in metastatic PCa patients (n = 10). The corresponding interventional procedures are demonstrated by schematic graphs alongside. All patients responded with a decline in serum PSA after either intervention. Data were normalized by the averaged fluorescence intensity of all enrolled patients in each interventional group before therapy. Statistical analysis was performed with the two-tailed Paired Samples *t*-test. **C** Temperature variations of PS and AgNIS upon 80 s of 200 W microwave irradiation. Inset: thermographic images of PS and AgNIS before and after microwave irradiation was applied. **D** Schematic showing the evaluation of microwave acceleration on AgNIS. Biotin-BSA and CF647-labeled SA were used as model probes. Non-specific binding of CF647-SA to the surface under microwave was used as the background control. **E** Effect of irradiation time with 200 W microwave on the signal-to-background ratio. Microwave-accelerated specific binding represented by increased MFI was found on both PS and AgNIS, but the optimal S/B ratio of AgNIS is approximately twofold of PS. Significantly less binding was seen in controls without microwave irradiation on PS and AgNIS. **F** Calibration curves of microwave-accelerated fluorescence immunoassays on PS and AgNIS

To advance the AgNIS platform for prognostic perspectives, we recruited a cohort (n = 13) with histopathologically-confirmed localized PCa on-site at SYSUCC, subject to RALRP by a Da Vinci XI surgical system after clinical evaluation. Two blood draws were taken at pre- and post-operative visits for individual patients. The total PSA levels prominently dropped after surgical interventions, with a therapeutic response rate of 100% (Fig. 6A). Another cohort (n = 10) diagnosed with unresectable metastatic PCa was admitted to the hospital to receive systemic hormone therapy and surveilled by eFIA. Significant declines in PSA were detected following treatment (Fig. 6B), corroborating the prognostic significance of AgNIS. The total assay time is a critical consideration in clinical practice to reduce the turnaround time and optimize the streamlined workflow. Metallic silver colloids have been proposed for MEF with accelerated kinetics of assays by microwaves [45]. With low-power microwaves applied, there was an approximate 13 °C elevation in temperature for AgNIS, distinctly higher than PS (Fig. 6C). The effect is construed by the photothermal effect of plasmonic AgNIS in response to microwave electromagnetic radiation. Such instantaneous heat shock can preserve the protein functionalities to a great extent. We studied the impact of microwaves on molecular interactions using the biotin-SA system (Fig. 6D). The interaction period was appreciably shortened to 80 s with ten fold specific binding at optimum (Fig. 6E). A comparable calibration curve could be established by microwave acceleration on the antigen recognition by dAb that outperformed conventional PS substrate (Fig. 6F). Thus, the entire duration of eFIA can be potentially curtailed to a considerable degree as an exclusive advantage of AgNIS in the future.

## Conclusions

In summary, we studied the redox effects of reducing monosaccharides on the synthesis of Ag structures under precisely controlled catalysis of AuNPs. We confirmed that potent multiplexed MEF originated from a well-balanced redox reaction at a stable reduction rate, making the most reducing saccharide glucose less optimal. The optimal nanostructure, namely AgNIS, was thoroughly characterized as semi-continuous in irregularity with a medium (instead of small or large) gap distance and sufficient abundance, inferred from gap distribution profiling. FDTD shows that the inhomogeneity afforded different electric field distribution contours at different wavelengths, accounting for the MEF in a broad spectrum. The platform presented strong stability at various pH and temperatures with all dyes (Additional file 1: Fig. S24). Modulated by BSA with a high DoL of biotins, a highly sensitive eFIA was conducted on AgNIS towards PSA detection in PCa cells, xenograft tumors, and patient sera at the convenience of a low sample volume required. The proposed assay does not change the current accustomed instruments and consumables and is accorded with clinical ECLIA results. The prognostic appraisal was validated in cohorts receiving RALRP and hormone therapy. We also provide preliminary MEF evidence in a point-of-care setup using a smartphone. Last but not least, we manifested that a microwave acceleration strategy can enormously abbreviate assay time. The strong diagnostic performance may eliminate the possibility of overdiagnosis and false negativity. Serial biomarker tracking during the entire course of therapeutic procedures and detection of potential biochemical recurrence need to be accomplished in the foreseeable future.

## Supplementary Information

**Additional file 1: Table S1**. Baseline characteristics of enrolled subjects in the study. **Table S2**. Comparison of LODs for ultrasensitive PSA detection. **Fig. S1**. Immobilization of AuNPs on PS substrate. **Fig. S2**. Comparison of reducing monosaccharides. **Fig. S3**. Cyclic voltammograms of monosaccharide oxidation. **Fig. S4**. Extinction spectra of Ag structures by galactose reduction. **Fig. S5**. Extinction spectra of Ag structures by glucose reduction. **Fig. S6**. Extinction spectra of Ag structures by mannose reduction. **Fig. S7**. Extinction spectra of Ag structures by xylose reduction. **Fig. S8**. Extinction spectra of Ag structures by arabinose reduction. **Fig. S9**. Covalent modification of BSA with various fluorophores. **Fig. S10**. Scree plot of eigenvalues for PCA analysis. **Fig. S11**. Surface morphologies of Ag structures synthesized by mannose. **Fig. S12**. Quantification of Ag surface coverage. **Fig. S13**. Measurement of the vertical dimension of AgNIS. **Fig. S14**. Broad spectral fluorescence enhancement by AgNIS. **Fig. S15**. Stability of the broad spectral fluorescence on AgNIS. **Fig. S16**. Gap distribution calculation for AgNIS. **Fig. S17**. Mannose-mediated synthesis of AgNPs. **Fig. S18**. Magnetron sputtered continuous Ag thin films. **Fig. S19**. AuNP seed-mediated synthesis of continuous Au films. **Fig. S20**. Surface hydrophobicity of AgNIS. **Fig. S21**. Characterization of the biotin labeling ratio. **Fig. S22**. Surface adsorption of BSA on AgNIS. **Fig. S23**. Correlation of PSA measured by ECLIA with ISUP grades. **Fig. S24**. Effects of pH and temperature on fluorescence signals.

## Data Availability

The authors declare that all data supporting the findings of this work are available within the paper and Supplementary Information. Source data for figures in the study are available in the *Research Data Deposit* (RDD) medical research platform with the identifier number RDDB2022631437.

## Code availability

The custom MATLAB codes associated with the calculation of gap distance distribution are available from the authors upon request.
