# Supplementary material for: Monosaccharide-mediated rational synthesis of a universal plasmonic platform with broad spectral fluorescence enhancement for high-sensitivity cancer biomarker analysis
Source: J Nanobiotechnology. 2022 Apr 10;20:184. doi: 10.1186/s12951-022-01359-z (PMC8995057; doi:10.1186/s12951-022-01359-z)
Supplement: Supplementary file 1 — Additional file 1: Table S1. Baseline characteristics of enrolled subjects in the study. Table S2. Comparison of LODs for ultrasensitive PSA detection. Fig. S1. Immobilization of AuNPs on PS substrate. Fig. S2. Comparison of reducing monosaccharides. Fig. S3. Cyclic voltammograms of monosaccharide oxidation. Fig. S4. Extinction spectra of Ag structures by galactose reduction. Fig. S5. Extinction spectra of Ag structures by glucose reduction. Fig. S6. Extinction spectra of Ag structures by mannose reduction. Fig. S7. Extinction spectra of Ag structures by xylose reduction. Fig. S8. Extinction spectra of Ag structures by arabinose reduction. Fig. S9. Covalent modification of BSA with various fluorophores. Fig. S10. Scree plot of eigenvalues for PCA analysis. Fig. S11. Surface morphologies of Ag structures synthesized by mannose. Fig. S12. Quantification of Ag surface coverage. Fig. S13. Measurement of the vertical dimension of AgNIS. Fig. S14. Broad spectral fluorescence enhancement by AgNIS. Fig. S15. Stability of the broad spectral fluorescence on AgNIS. Fig. S16. Gap distribution calculation for AgNIS. Fig. S17. Mannose-mediated synthesis of AgNPs. Fig. S18. Magnetron sputtered continuous Ag thin films. Fig. S19. AuNP seed-mediated synthesis of continuous Au films. Fig. S20. Surface hydrophobicity of AgNIS. Fig. S21. Characterization of the biotin labeling ratio. Fig. S22. Surface adsorption of BSA on AgNIS. Fig. S23. Correlation of PSA measured by ECLIA with ISUP grades. Fig. S24. Effects of pH and temperature on fluorescence signals. [file 12951_2022_1359_MOESM1_ESM.docx]

**Supplementary Information**

**Monosaccharide-mediated rational synthesis of a universal plasmonic platform with broad spectral fluorescence enhancement for high-sensitivity cancer biomarker analysis**

Mengyao Liu^1,#^, Yonghong Li^1,2,#^, Wei Xing^1,3,^*, Yuqin Zhang^4^, Xi Xie^5^, Jiadong Pang^1^, Fangjian Zhou^1,2^, Jiang Yang^1,^*

^1^State Key Laboratory of Oncology in South China, Collaborative Innovation Center for Cancer Medicine, Sun Yat-sen University Cancer Center, Guangzhou, China

^2^Department of Urology, Sun Yat-sen University Cancer Center, Guangzhou, China

^3^Department of Anesthesiology, Sun Yat-sen University Cancer Center, Guangzhou, China

^4^School of Automation, Hangzhou Dianzi University, Hangzhou, China

^5^State Key Laboratory of Optoelectronic Materials and Technologies, School of Electronics and Information Technology, Sun Yat-sen University, Guangzhou, China

^#^These authors contributed equally

*Correspondence: [yangjiang@sysucc.org.cn](mailto:yangjiang@sysucc.org.cn) or xingwei@sysucc.org.cn

**Table of Contents**

**Supplementary information**

Supplementary Table 1 | Baseline characteristics of enrolled subjects in the study……………..3

Supplementary Table 2 | Comparison of LODs for ultrasensitive PSA detection………………3

Supplementary Fig. 1 | Immobilization of AuNPs on PS substrate………………………………..4

Supplementary Fig. 2 | Comparison of reducing monosaccharides………………………………5

Supplementary Fig. 3 | Cyclic voltammograms of monosaccharide oxidation…………………..6

Supplementary Fig. 4 | Extinction spectra of Ag structures by galactose reduction……………..7

Supplementary Fig. 5 | Extinction spectra of Ag structures by glucose reduction……………….8

Supplementary Fig. 6 | Extinction spectra of Ag structures by mannose reduction……………..9

Supplementary Fig. 7 | Extinction spectra of Ag structures by xylose reduction……………….10

Supplementary Fig. 8 | Extinction spectra of Ag structures by arabinose reduction……………11

Supplementary Fig. 9 | Covalent modification of BSA with various fluorophores…..…………..12

Supplementary Fig. 10 | Scree plot of eigenvalues for PCA analysis…..……………...………..12

Supplementary Fig. 11 | Surface morphologies of Ag structures synthesized by mannose…..13

Supplementary Fig. 12 | Quantification of Ag surface coverage…………………………………14

Supplementary Fig. 13 | Measurement of the vertical dimension of AgNIS……………………14

Supplementary Fig. 14 | Broad spectral fluorescence enhancement by AgNIS……………..…15

Supplementary Fig. 15 | Stability of the broad spectral fluorescence on AgNIS……………..…16

Supplementary Fig. 16 | Gap distribution calculation for AgNIS……………..…………………..17

Supplementary Fig. 17 | Mannose-mediated synthesis of AgNPs……………..……………….18

Supplementary Fig. 18 | Magnetron sputtered continuous Ag thin films……………..…………19

Supplementary Fig. 19 | AuNP seed-mediated synthesis of continuous Au films…………..…19

Supplementary Fig. 20 | Surface hydrophobicity of AgNIS…………..…………………………..20

Supplementary Fig. 21 | Characterization of the biotin labeling ratio……………………………21

Supplementary Fig. 22 | Surface adsorption of BSA on AgNIS…………………………………..22

Supplementary Fig. 23 | Correlation of PSA measured by ECLIA with ISUP grades…………..22

Supplementary Fig. 24 | Effects of pH and temperature on fluorescence signals. ……………23

Supplementary references…………………………….……………………………………………24

**Supplementary Information**


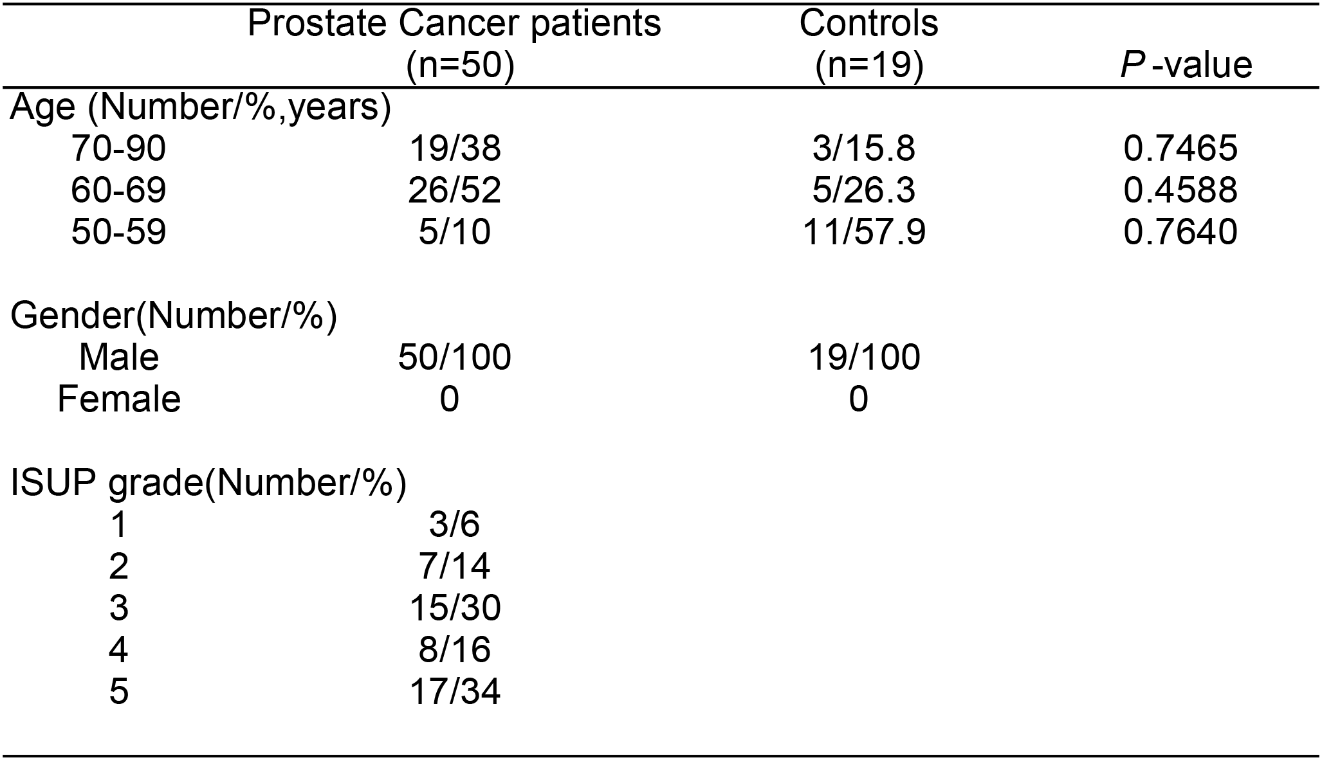


**Supplementary Table 1 | Baseline characteristics of prostate cancer (PCa) patients and healthy subjects screened in the study.**

**
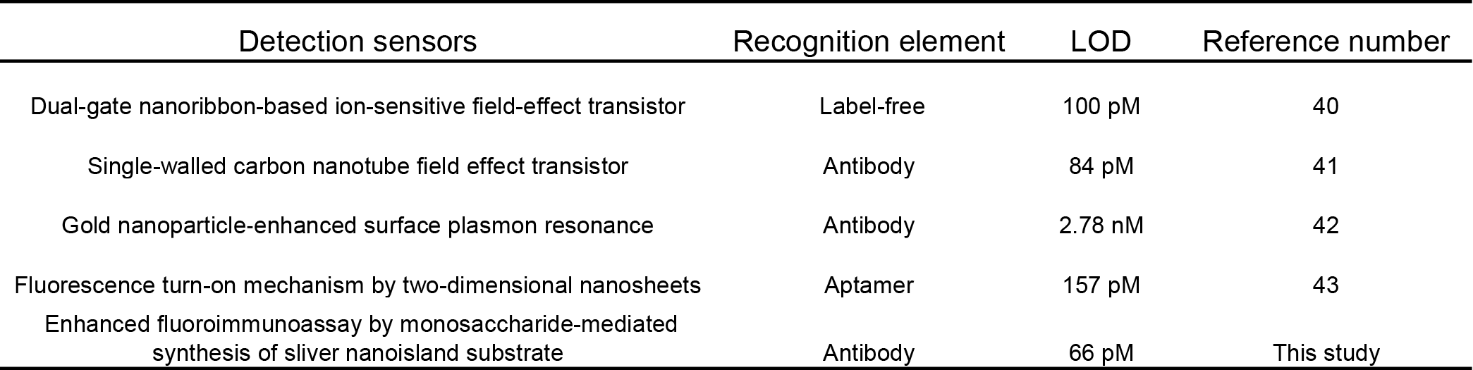
**

**Supplementary Table 2 | Comparison of limits of detection (LODs) for ultrasensitive PSA detection**


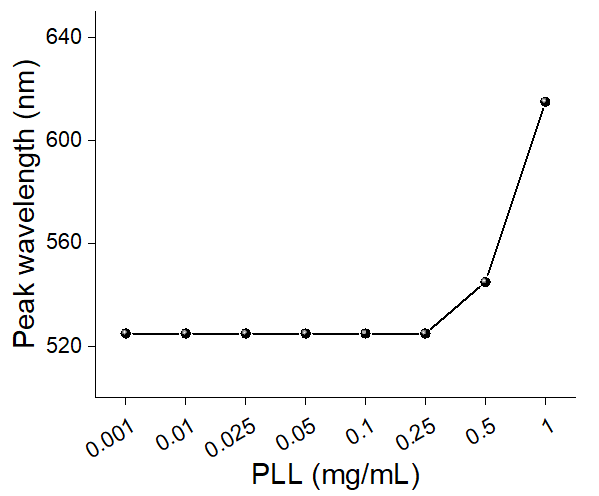


**Supplementary Fig. 1 | Immobilization of gold nanoparticles (AuNPs) on polystyrene (PS) surface through electrostatic interactions as seeds for catalytic growth of silver nanostructures.** The peak wavelength of localized surface plasmon resonance (LSPR) for AuNPs immobilized on PS surface modified with gradient concentrations of PLL. The peak remained at 525 nm as colloid AuNPs at PLL concentrations up to 0.25 mg mL^-1^, while it shifted bathochromically above this threshold concentration. Data points represent triplicates, all of which have exactly the same wavelengths.


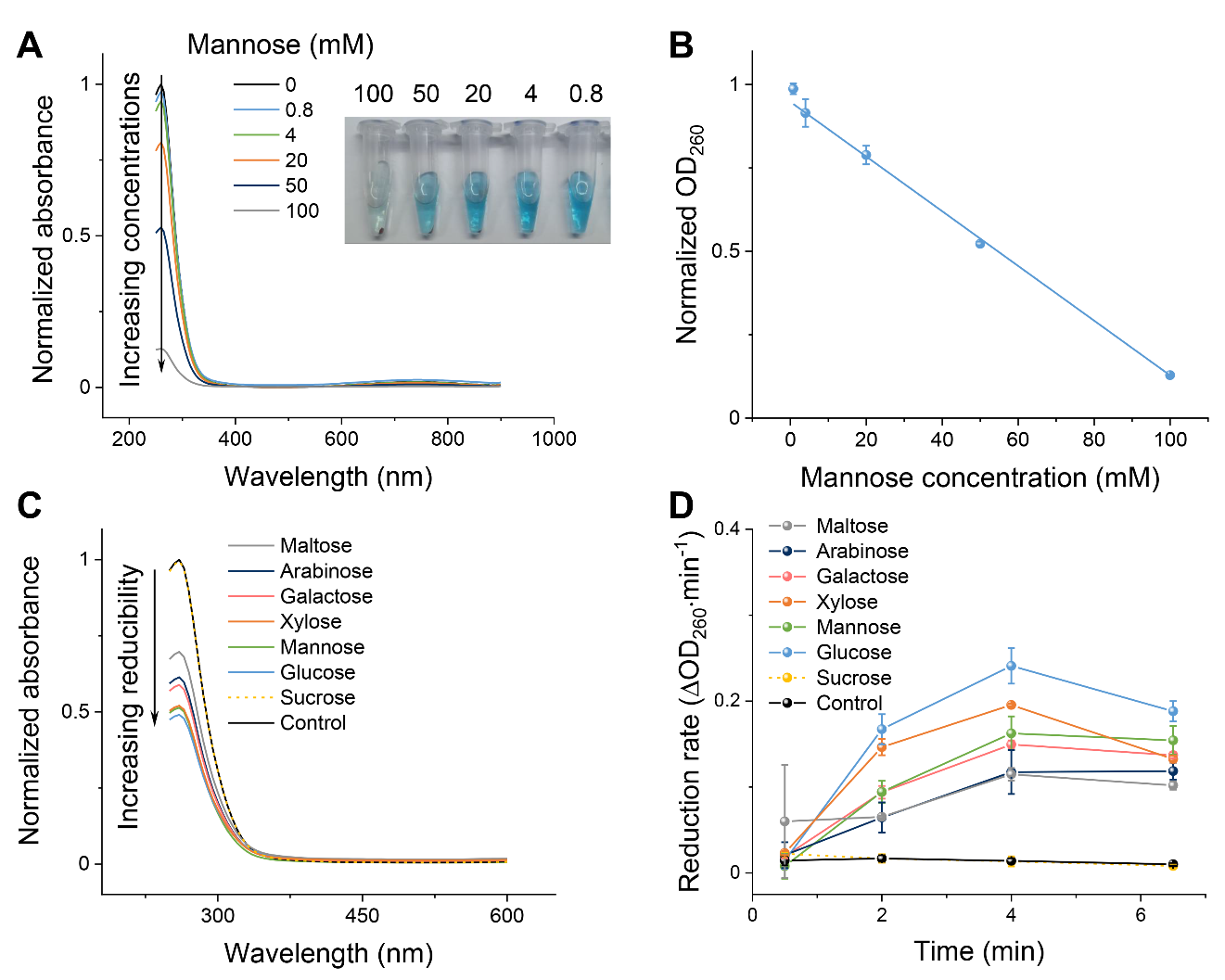


**Supplementary Fig. 2 | Comparison of reducing monosaccharides by Benedict’s assay. (A)** UV-vis absorption spectra of Benedict’s solution in the presence of a mannose concentration gradient. Signature absorption at 260 nm decreases with increasing concentrations of mannose, indicated by the arrow. Inset shows the corresponding photograph of Benedict’s mixtures. **(B)** Correlation of the optical density at 260 nm with mannose concentrations. Since the redox reaction involving aldehyde oxidation into carboxylates by Cu^2+^ under basic conditions is nonstoichiometric, the dynamic working range was identified between 4 and 100 mM with a linear fit (R=0.998). **(C)** Absorption spectra of Benedict’s assay for 50 mM various monosaccharides. The non-reducing disaccharide sucrose was used as a control along with an aqueous control without saccharides. Data were normalized by OD_260_ of the respective mixtures before redox reactions. **(D)** The average reduction rate of individual monosaccharides was monitored and calculated as variations of OD_260_ per min. Glucose and xylose exhibited elevated reduction rates at first, which then rapidly declined, while mannose maintained a relatively stable rate of reduction throughout as well as an adequate end-point reduction.


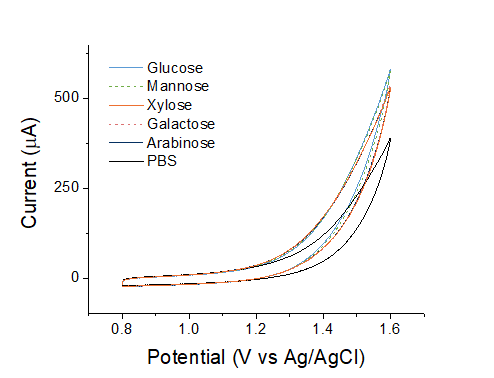


**Supplementary Fig. 3 | Cyclic voltammograms of monosaccharide oxidation.** Higher oxidation currents are suggestive of stronger reducing properties. The scan was performed in 0.1 M pH 7.0 PBS in the presence of 10 mM monosaccharides in the potential window of 0.8-1.6 V at a scan rate of 0.1 V s^-1^.

**
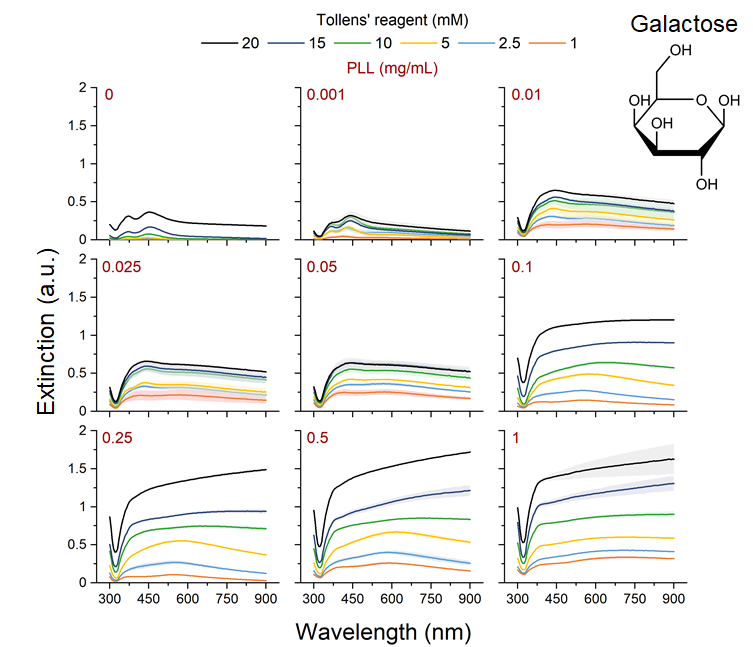
**

**Supplementary Fig. 4 | Extinction spectra of metallic Ag structures synthesized under a variety of conditions with galactose.** Concentration gradients of poly-l-lysine (PLL) and the Tollens’ reagent covered a matrix of 0 to 1 mg mL^-1^ and 1 to 20 mM, respectively. Each synthesis condition gave rise to unique extinction properties. In general, extinction increases with higher PLL and the Tollens’ reagent concentrations**,** increasing thicknesses and coalescence into bulk structures. Galactose was kept at 10 mM. Shaded areas indicate corresponding 95% confidence intervals for independent triplicates.

**
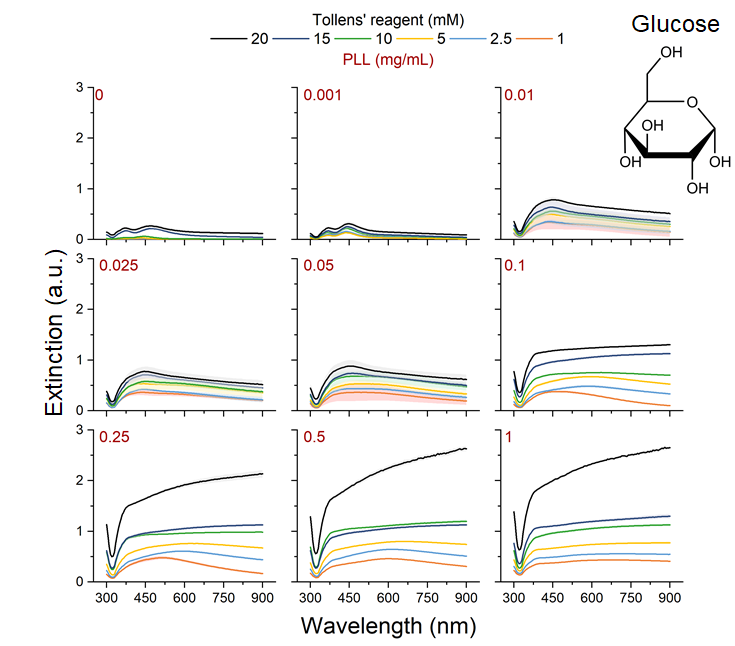
**

**Supplementary Fig. 5 | Extinction spectra of metallic Ag structures synthesized under a variety of conditions with glucose.**

**
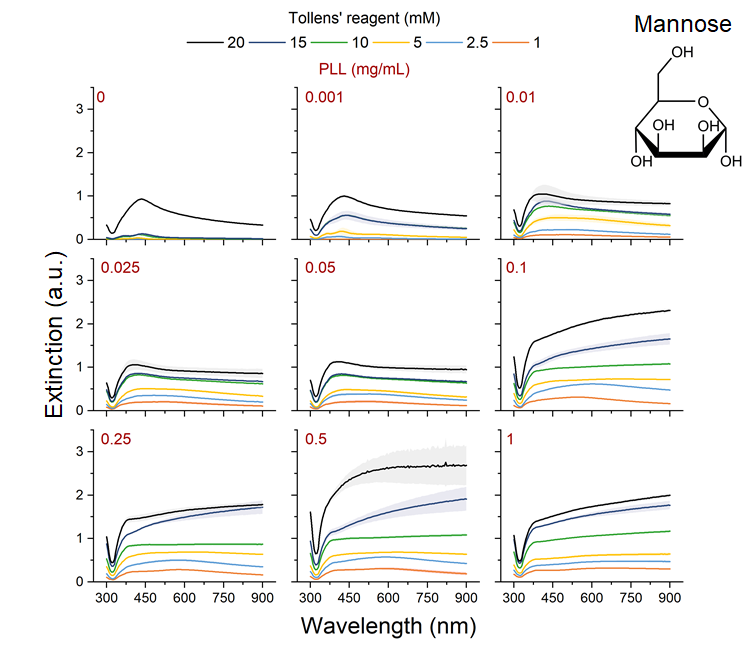
**

**Supplementary Fig. 6 | Extinction spectra of metallic Ag structures synthesized under a variety of conditions with mannose.**

**
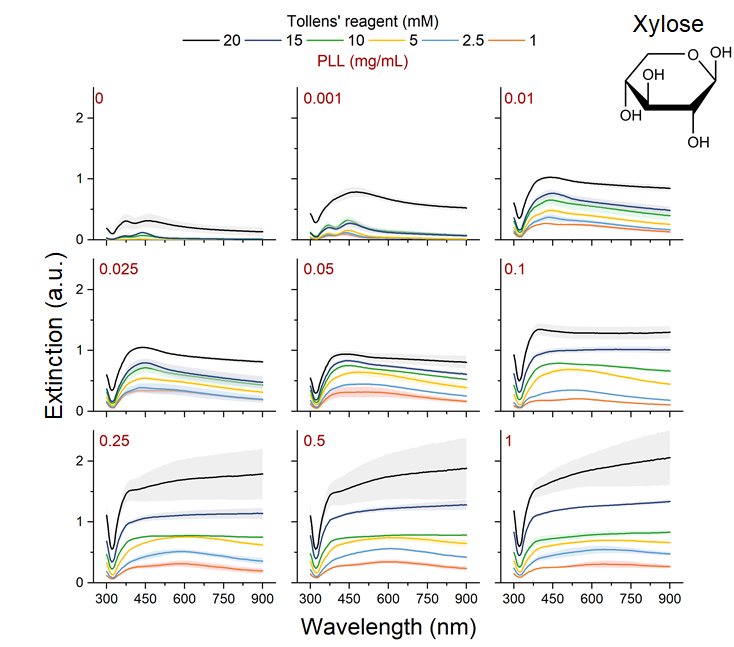
**

**Supplementary Fig. 7 | Extinction spectra of metallic Ag structures synthesized under a variety of conditions with xylose.**

**
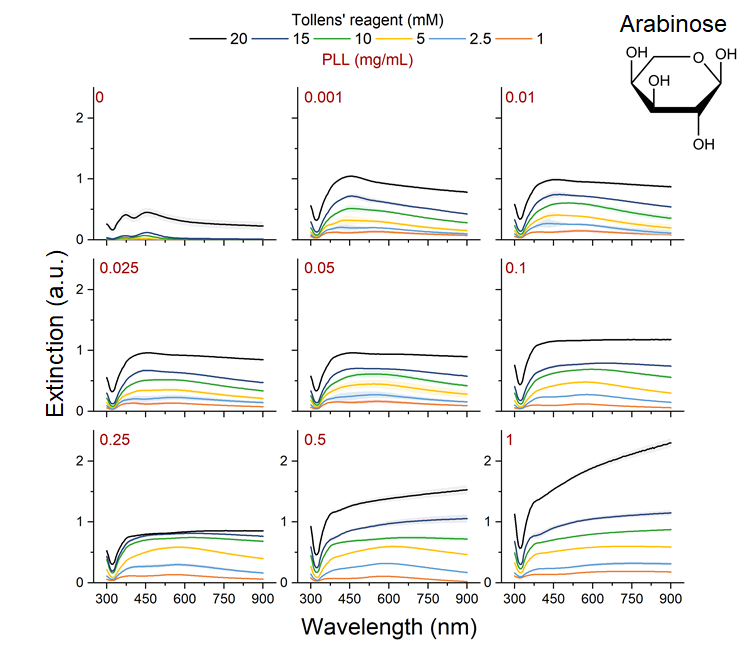
**

**Supplementary Fig. 8 | Extinction spectra of metallic Ag structures synthesized under a variety of conditions with arabinose.**

**
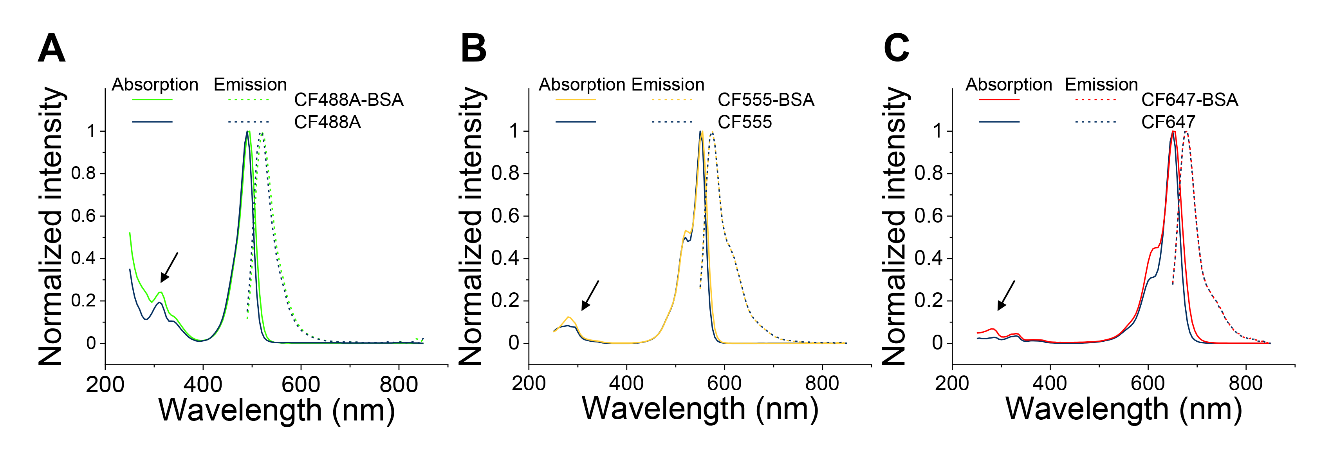
Supplementary Fig. 9 | Covalent modification of BSA with various fluorophores of CF488A, CF555, and CF647 by the NHS-amine chemistry.** UV-vis absorption (solid lines) and fluorescence emission spectra (dotted lines) of **(A)** CF488A, **(B)** CF555, and **(C)** CF647 before and after covalent conjugation with BSA. After purification, an increase in the characteristic protein absorption of BSA at 280 nm was observed, as indicated by arrows, implying successful crosslinking. The labeling ratios of dyes to BSA were calculated to be 4.15, 5.88, and 4.82, from the changes in OD_280_ and the peak absorption of corresponding fluorophores based on molar extinction coefficients of 70000, 150000, 240000, and 43824 cm^-1^ M^-1^ for CF488A, CF555, CF647, and BSA, respectively.


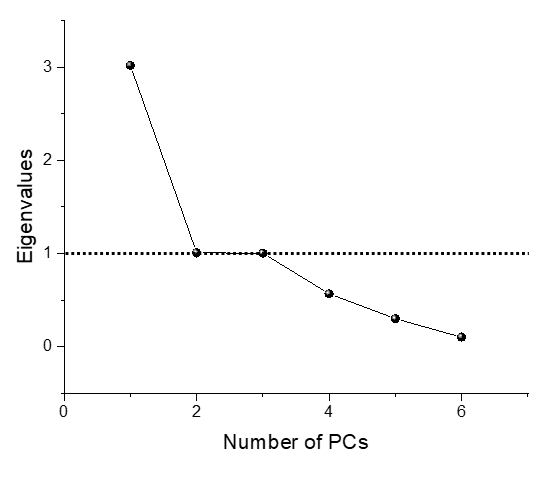


**Supplementary Fig. 10 | Scree plot of eigenvalues for each principal component.** Principal component analysis was performed on a correlation matrix of synthesis conditions and the fluorescence enhancement at different wavelengths. The “Kaiser-Guttman rule” criterion that defines the cut-off for PC retention (eigenvalue >1) is indicated by the dotted line.

**
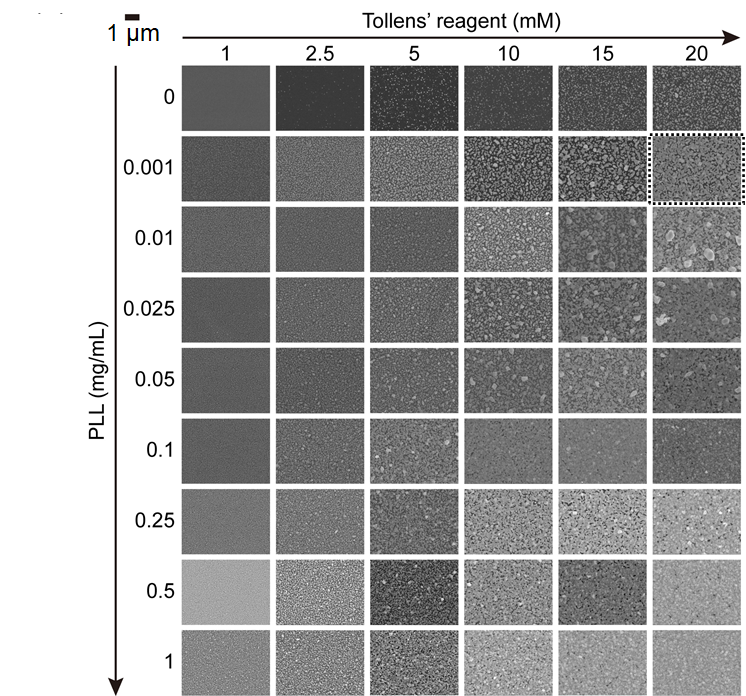
**

**Supplementary Fig. 11 | Surface morphologies visualized by SEM.** Silver structures were synthesized at concentration gradients of PLL and Tollens’ reagents with 10 mM mannose as the reducing agent. In the absence of catalytic AuNPs, only sparsely dispersed AgNPs formed, and the overall synthesis was inefficient, revealing the critical role of AuNP catalysts. The diverse synthesis conditions produced diversified structures, including discontinuous particles of various sizes and shapes, semi-continuous islands, and seamless continuous films. As expected, high concentrations of PLL and Tollens’ reagents led to the formation of Ag structures in increased continuity and coalescence, even with overgrown microscaled particles on top. The dotted boxed image is the structure with the optimal MEF for far-red CF647.

**
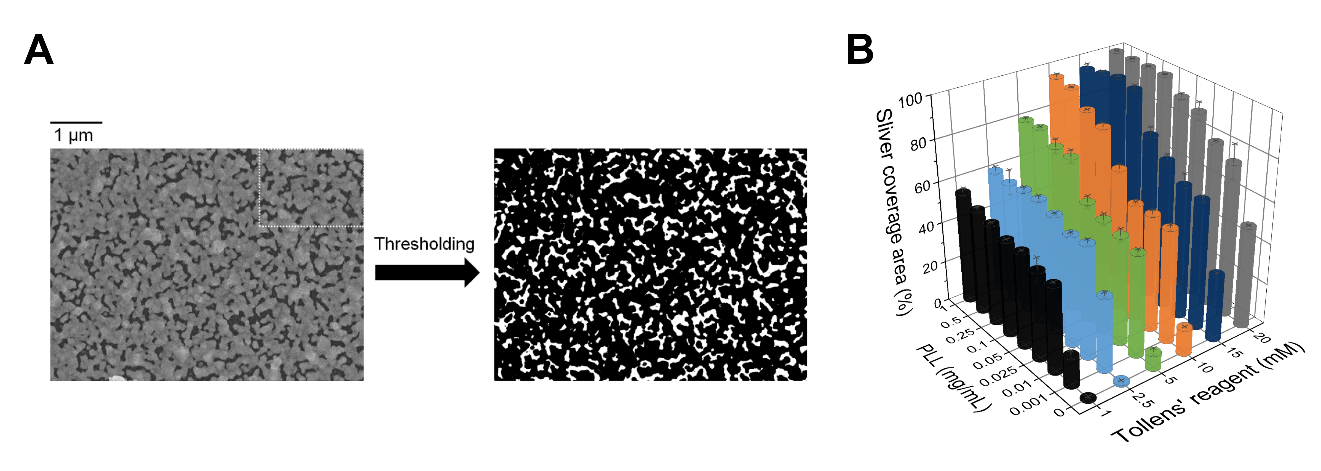
**

**Supplementary Fig. 12 | Quantification of silver surface coverage by color thresholding. (A)** For each SEM image of silver structures synthesized in different conditions (Fig. S11), 3 fields of view were selected for quantification. Automated thresholding was performed in the Image J software (NIH) to deconvolute black (silver) and white (gaps) areas. The relative silver coverage was calculated as black area/(black + white areas)*100%. An example SEM image (dotted box from Fig. S11) was the optimal synthesis condition of AgNIS by mannose with superior overall MEF for all colors (and optimal for CF647) and quantified to be 76% metallic silver coverage. The white boxed area was the region adopted for FDTD simulation (Fig. 2D). **(B)** The correlation of surface silver coverage with concentrations of PLL and the Tollens’ reagent.

**
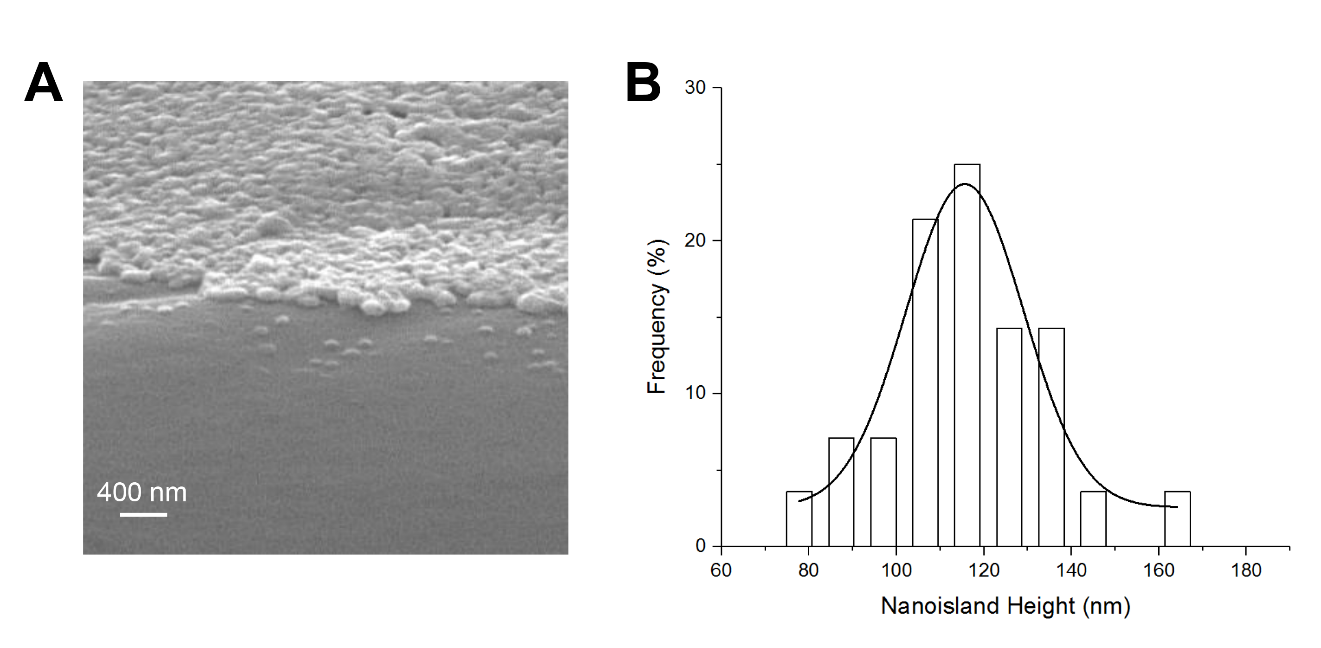
**

**Supplementary Fig. 13 | Measurement of the vertical dimension of AgNIS. (A)** An angled SEM image showing the height of AgNIS. **(B)** The height distribution profile of AgNIS showing an averaged vertical dimension of 116.4 ± 20.5 nm.


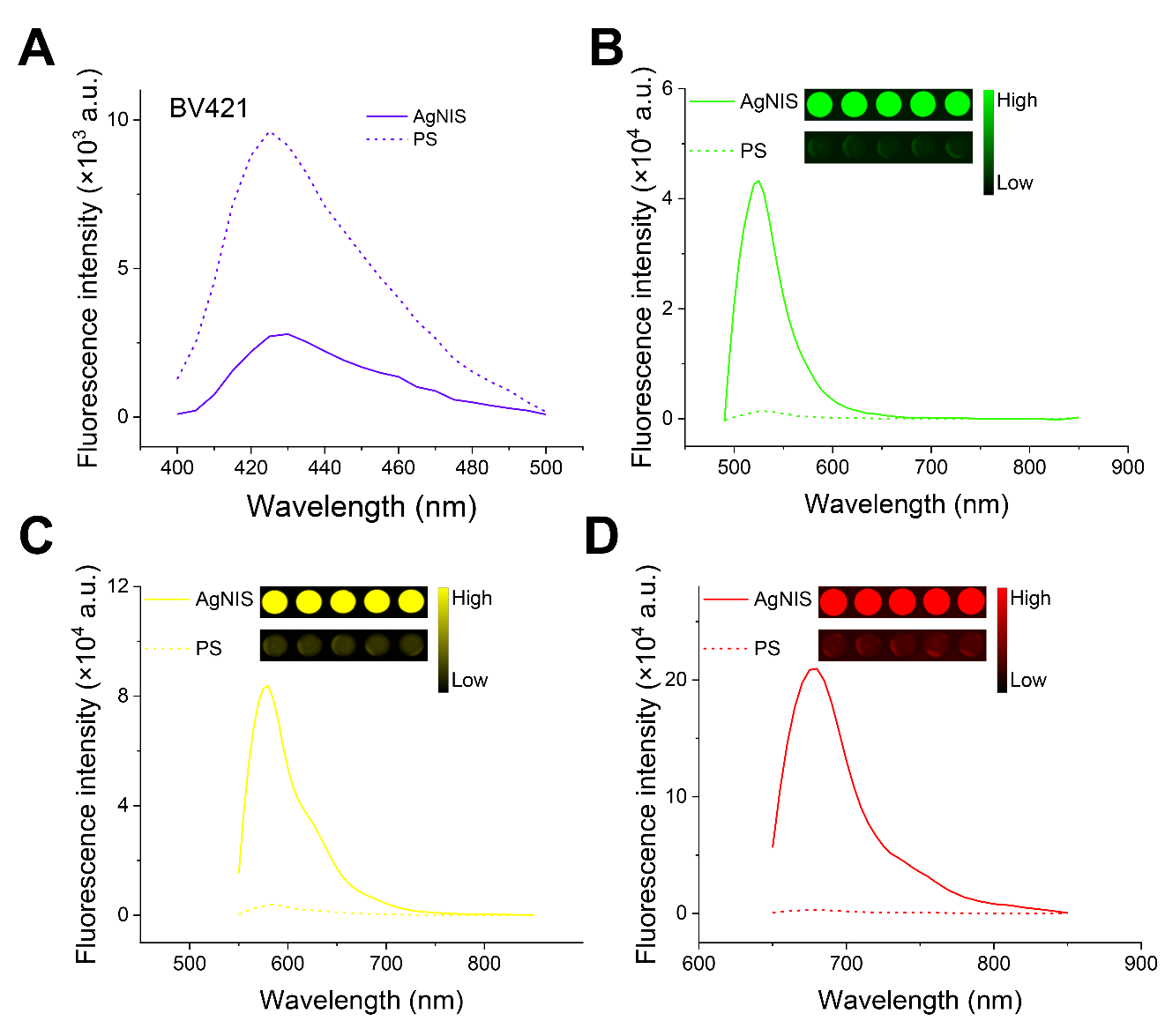


**Supplementary Fig. 14 | Broad spectral fluorescence enhancement by AgNIS.** Fluorescence emission spectra for covalently-labeled fluorophores of **(a)** BV421**, (b)** CF488A, **(c)** CF555, and **(d)** CF647 on PS (dashed lines) and AgNIS (solid lines) substrates. Insets show the spectrum-matched fluorescence images of CF488A-, CF555-, and CF647-labeled BSA complexes on PS and AgNIS, respectively. BV421 was an IgG conjugate in slightly larger dimensions of 14.5 nm x 8.5 nm x 4.0 nm, theoretically more favorable for MEF than smaller BSA at 14.0 nm x 4.0 nm x 4.0 nm^1,2^. Still, strong fluorescence quenching by AgNIS was observed instead. On the contrary, significant MEF of all other fluorophores in the visible-far red broad-spectrum was easily observable.


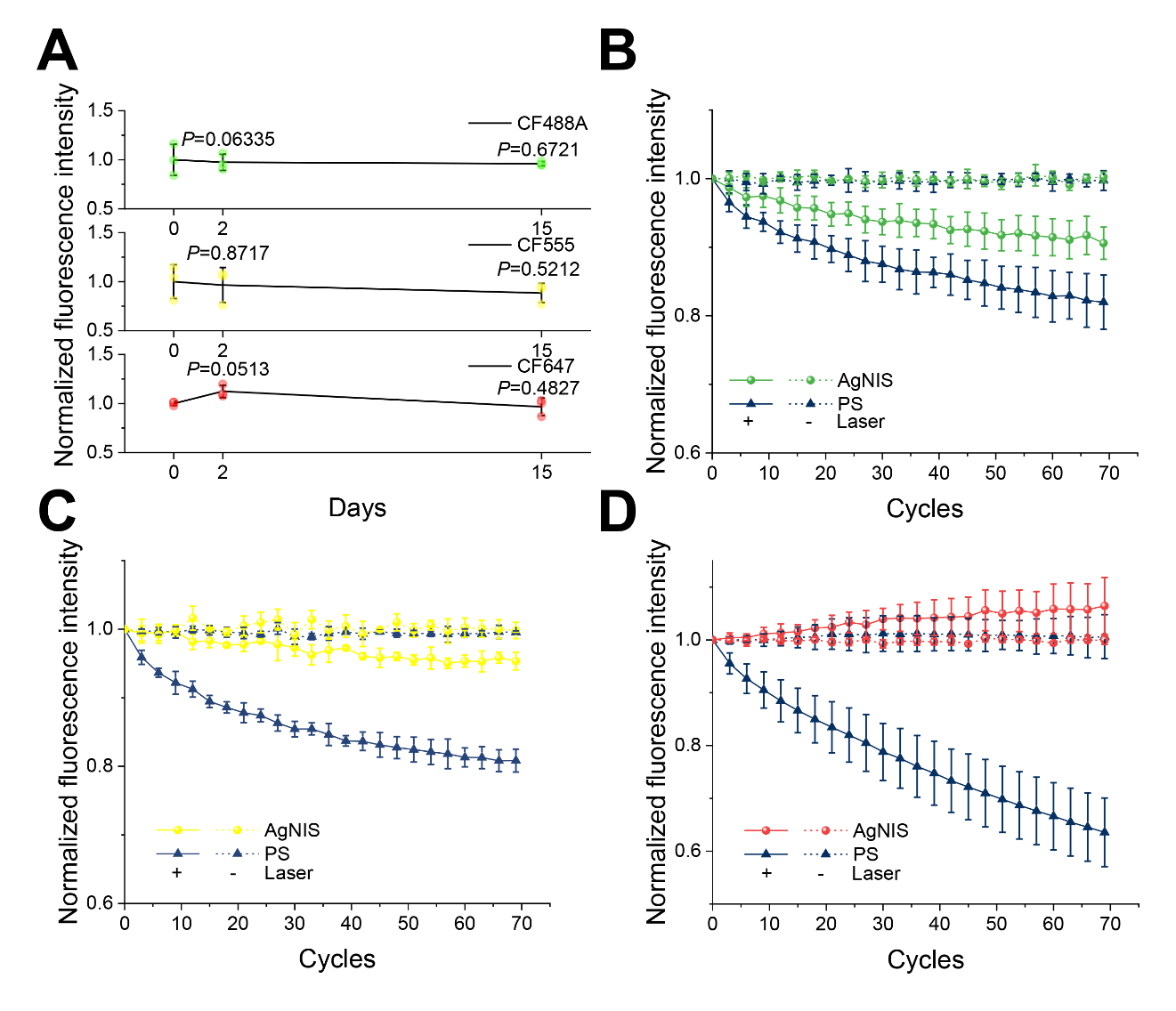


**Supplementary Fig. 15 | Stability of the broad spectral fluorescence on AgNIS. (A)** Long-term MEF stability of AgNIS. To maximally prevent ambient air oxidation, AgNIS was stored in vacuum at 4 ˚C for 2 and 15 days and incubated in 10 nM fluorescent BSA for 2 h at 4 ˚C. No statistical significance was found by a two-tailed Student’s *t*-test. Fluorescence intensity change of **(B)** CF488A, **(C)** CF555, and **(D)** CF647 on PS (triangles) and AgNIS (spheres) with (+) or without (-) excitation lasers, after overnight incubation in 1 μM covalently-labeled BSA probes. In the absence of lasers, fluorescence was sufficiently preserved on both PS and AgNIS, while the photostability of all fluorophores against intermittently cycled exposures was tremendously improved on AgNIS.


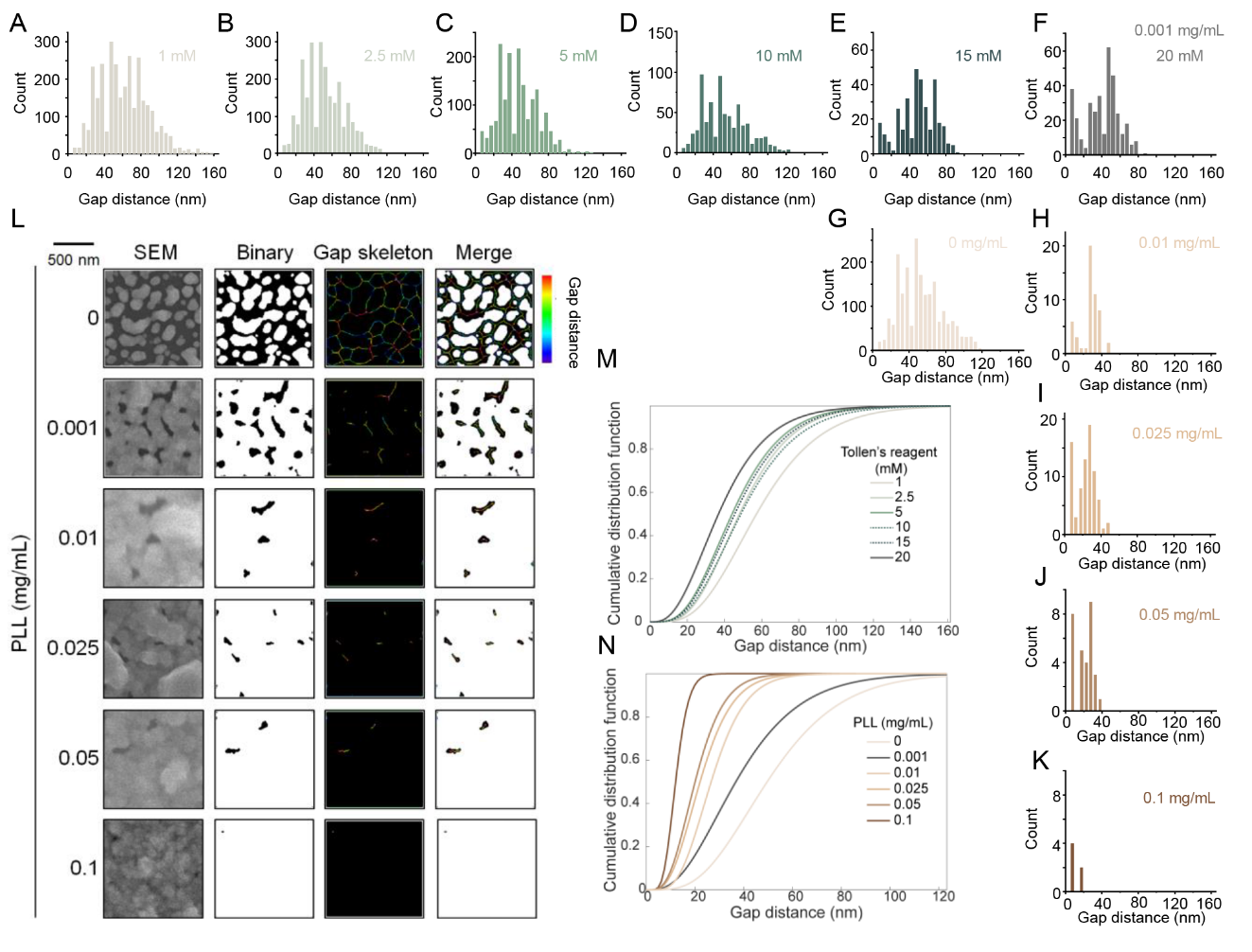


**Supplementary Fig. 16 | Gap distribution of AgNIS.** Individual gap distribution histograms of counts for silver structures synthesized at **(A)** 1, **(B)** 2.5, **(C)** 5, **(D)** 10, **(E)** 15, and **(F)** 20 mM Tollens’ reagent and a fixed 0.001 mg mL^-1^ PLL, or at **(G)** 0, **(F)** 0.001, **(H)** 0.01, **(I)** 0.025, **(J)** 0.05, and **(K)** 0.1 mg mL^-1^ PLL and a fixed 20 mM Tollens’ reagent. These synthesis conditions essentially control the characteristics of gaps. The gamma distribution function was used to fit the histograms^3^ to derive Fig. 2G-H. Despite that dispersed structures of NPs and discontinuous islands yield more gaps (Fig. 2F and S16A-F), they are large and less effective for MEF. As the growth concentration increases, the gap distance decreases but has a slight retracement at 10 mM (Fig. 2G) due to agglomeration and formation of large discontinuous islands that are less optimal for MEF than semi-continuous counterparts. In the light of catalyst seeding densities, the absence of PLL results in NP formation with large inefficient gaps. In contrast, high PLL concentrations with structural continuity produce smaller than optimal gaps but in deficiency (low counts). In addition, gaps smaller than a certain threshold value may be unable to position adsorbed BSA molecules (the largest dimension at ~14 nm) within the gaps, switching to the much weaker surface-enhancement mode due to weaker electric fields on the surface than in gaps (Fig. 2D). **(L)** SEM image-based calculation processes of gap distribution for silver structures synthesized at 0 to 0.1 mg mL^-1^ PLL and a fixed Tollens’ reagent of 20 mM. Midpoints of the nearest two pixels from an indefinite gap constituted the skeleton contour with the distance of gaps denoted in color scale. Cumulative distribution profiles for silver structures synthesized at **(M)** fixed PLL or **(N)** Tollens’ reagents. In general, as the PLL and Ag growth concentrations increase, the gap distribution shifts towards the smaller gap distance in higher probabilities. The best MEF condition (black lines in **M** and **N**) practically has the most “efficient” gaps for MEF in medium distances and sufficient abundance. It should be sufficiently large to host gap-situated BSA and sufficiently small for potent MEF (but not in direct contact), which can only be realized by semi-continuity rather than complete continuity and discontinuity.


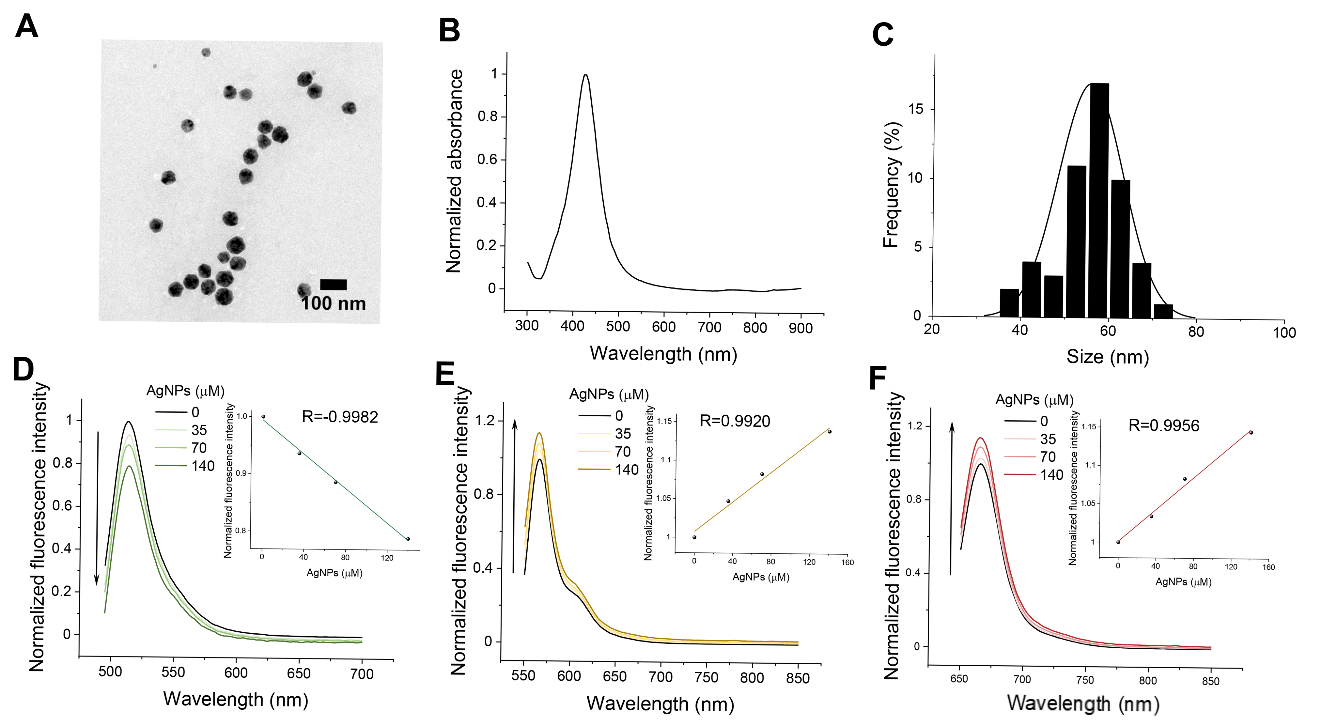


**Supplementary Fig. 17 | Mannose-mediated synthesis of AgNPs and the effects on different fluorophores. (A)** A representative TEM image, **(B)** the UV-vis absorption spectrum, and **(C)** the hydrodynamic size of AgNPs synthesized through reduction by mannose. The particle size was calculated to be 55.8±7.4 nm. Fluorescence spectra for **(D)** CF488A-, **(E)** CF555-, and **(F)** CF647-labeled BSA in the presence of gradient concentrations of AgNPs. Insets: peak fluorescence intensity as a function of the concentration of AgNPs. While the fluorescence enhancement for CF555 and CF647 of longer wavelengths was slightly enhanced by less than 20%, CF488A fluorescence was quenched.


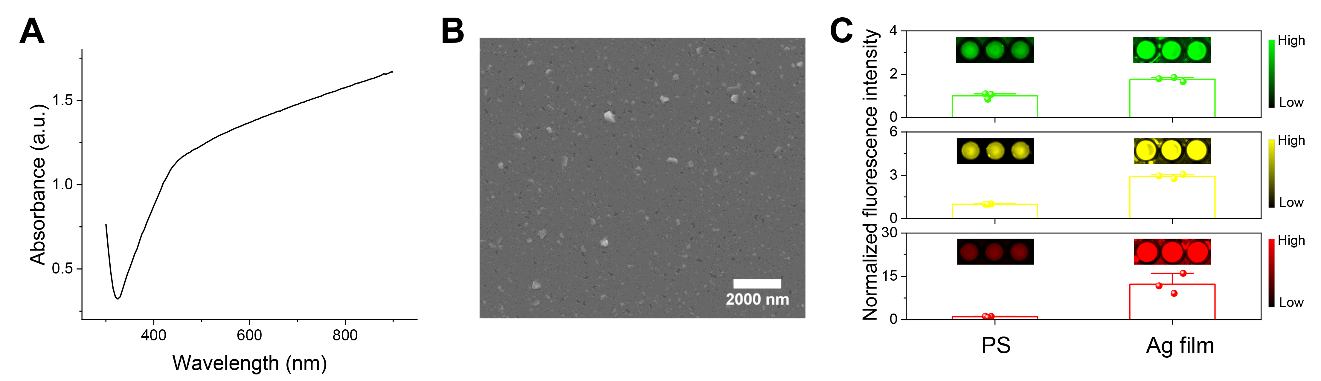


**Supplementary Fig. 18 | Continuous Ag thin films prepared by magnetron sputtering. (A)** The UV-vis absorption spectrum and **(B)** the SEM image of the magnetron-sputtered Ag film. Strong light absorption was seen in the visible region starting from approximately 450 nm, with even enhanced near-infrared (NIR) light absorption. The film is homogeneous with no apparent cracks, and a few dispersed small crystallites can be seen on the surface. **(C)** Fluorescence of CF488A-, CF555-, and CF647-labeled BSA probes on PS and magnetron-sputtered Ag films. Ag films were immersed in 100 nM probes for 2 h incubation at room temperature. The enhancement of all fluorophores on Ag films is somewhat limited except CF647 and is weaker than AgNIS.


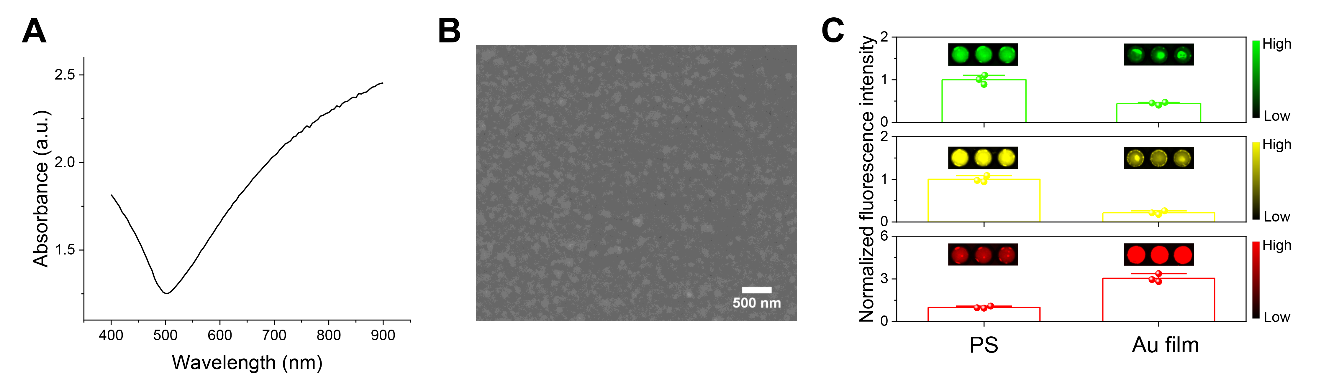


**Supplementary Fig. 19 | Solution-phase synthesis of continuous Au films with AuNP seed-mediated growth. (A)** The UV-vis absorption spectrum and **(B)** the SEM image of the synthesized Au film. Au films were synthesized similarly by seeding AuNPs as catalysts and autonomously grown in a 10 mM equimolar solution of HAuCl_4_ and hydroxylamine. The spectral absorption features are analogous to sputtered Ag films (Fig. S18) and evaporated Au films, with significant absorption in the NIR region. The Au surface coverage is complete and homogeneous. **(C)** Fluorescence of CF488A-, CF555-, and CF647-labeled BSA probes on PS and Au films. Conversely to sputtered Ag films and AgNIS, CF488A and CF555 are considerably quenched, and the fluorescence of CF647 is vaguely enhanced.


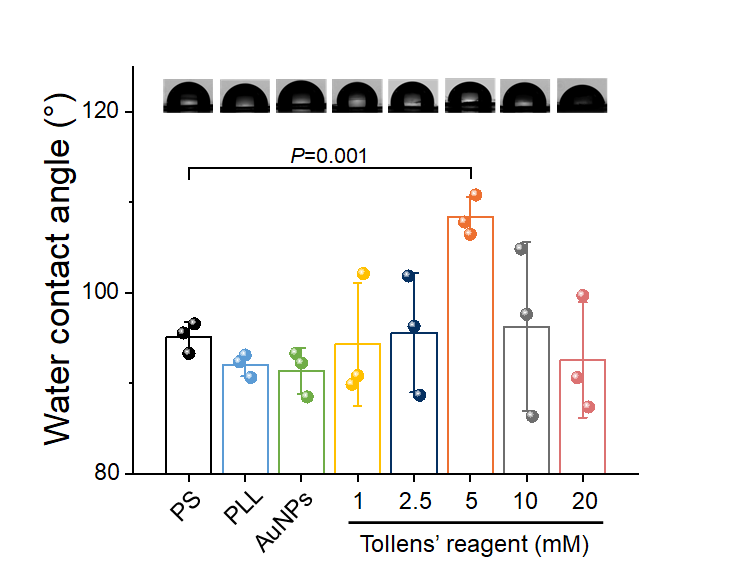


**Supplementary Fig. 20 | Surface hydrophobicity of AgNIS measured by water contact angles.** The PLL concentrations in all syntheses were kept at the optimum of 0.001 mg mL^-1^. PS retained its intrinsic hydrophobicity (statistically insignificant decrease) after modification with hydrophilic PLL and AuNPs, due to the low PLL concentration and thus a low seeding density of AuNPs (Fig. 1C). The water contact angles first insignificantly increased with increasing growth concentrations and then decreased. The only statistical significance was at 5 mM Tollens’ reagent with a minor variation of ~15˚.


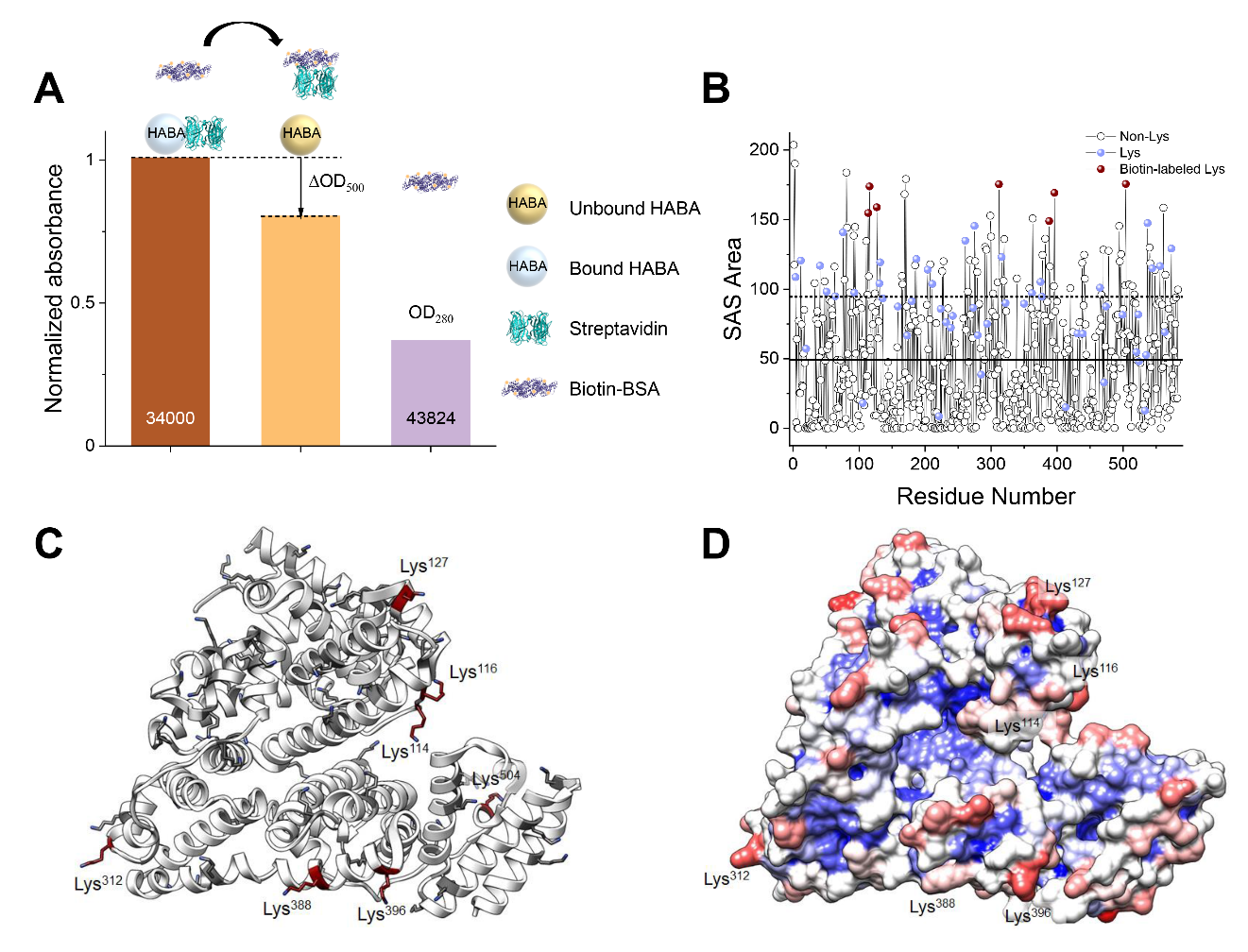


**Supplementary Fig. 21 | Characterization of the biotin labeling ratio. (A)** Colorimetric assay for estimation of biotin-BSA by 4'-hydroxyazobenzene-2-carboxylic acid (HABA). HABA molecules in the HABA- streptavidin (SA) complex, which intensely absorbs at 500 nm, are kinetically replaced by biotin-BSA in the competition, owing to stronger biotin-SA affinities. The decrease in OD500 was measured to derive the biotin concentration indirectly. The molarity of BSA was calculated accordingly after compensation for biotin absorbance at 280 nm. Extinction coefficient of HABA-SA complexes: 34,000 cm^-1^ M^-1^. The biotin-to-BSA ratio was calculated to be 7.11. **(B)** Solvent-accessible surface (SAS) area of amino acid (AA) residues in BSA (PDB: 4F5S). The 7 most accessible Lys residues out of 59 for amine side-chain reactions were identified as Lys504, Lys312, Lys116, Lys396, Lys127 Lys^114^, and Lys^388^ (brown), while other less accessible Lys residues are in light blue. Solid line: averaged SAS area of all AAs. Dotted line: above which all Lys residues are considered sterically available for conjugation. **(C)** Location of biotins in the BSA structure. **(D)** Surface mapping of SAS for BSA. High and low solvent accessibilities are shown in red and blue, respectively. The theoretical distribution of biotins is evenly across the entire BSA molecule.


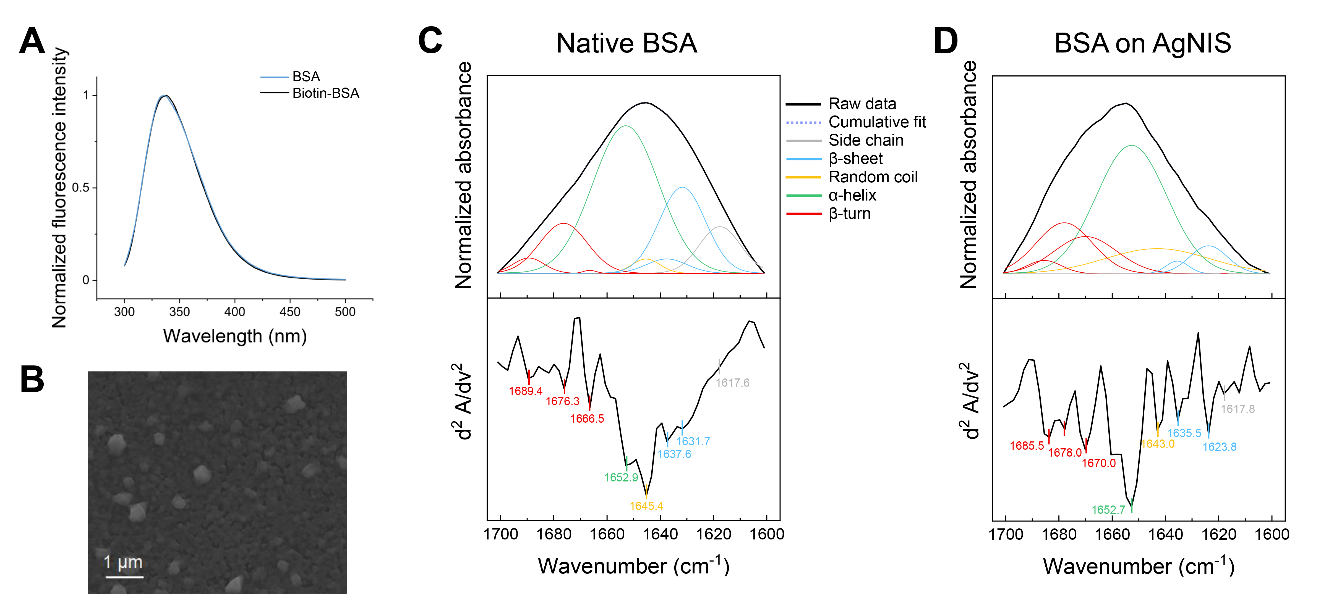


**Supplementary Fig. 22 | Surface adsorption of BSA on AgNIS. (A)** Steady-state intrinsic Trp fluorescence spectra of 5 μM BSA before and after biotinylation, excited at 295 nm. No apparent peak shifts were observed. **(B)** A top-view SEM image of adsorbed biotin-BSA on AgNIS. Aggregated protein molecules can be seen. Attenuated total reflection-Fourier transform infrared (ATR-FTIR) spectra (upper panels) of **(C)** native BSA and **(D)** BSA adsorbed on AgNIS in the amide I region. Intense protein absorption bands can be observed between 1600 and 1700 cm^-1^, primarily governed by C=O stretching vibrations. Second derivative spectra are shown at the bottom to resolve secondary structures by peak deconvolution, following reported band assignments^4, 5^.


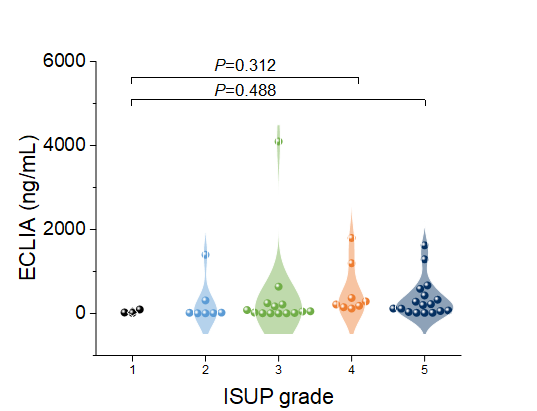


**Supplementary Fig. 23 | Violin plot showing the correlation of serum PSA clinically measured by ECLIA with ISUP grades.** Patients were stratified based on Gleason Scores according to the International Society of Urological Pathology grading, identical as in Fig. 5K. Statistical analysis was performed by one-way analysis of variance (ANOVA) with Fisher's Least Significant Difference (LSD) Test. No statistical significance was found between ISUP grades using ECLIA.


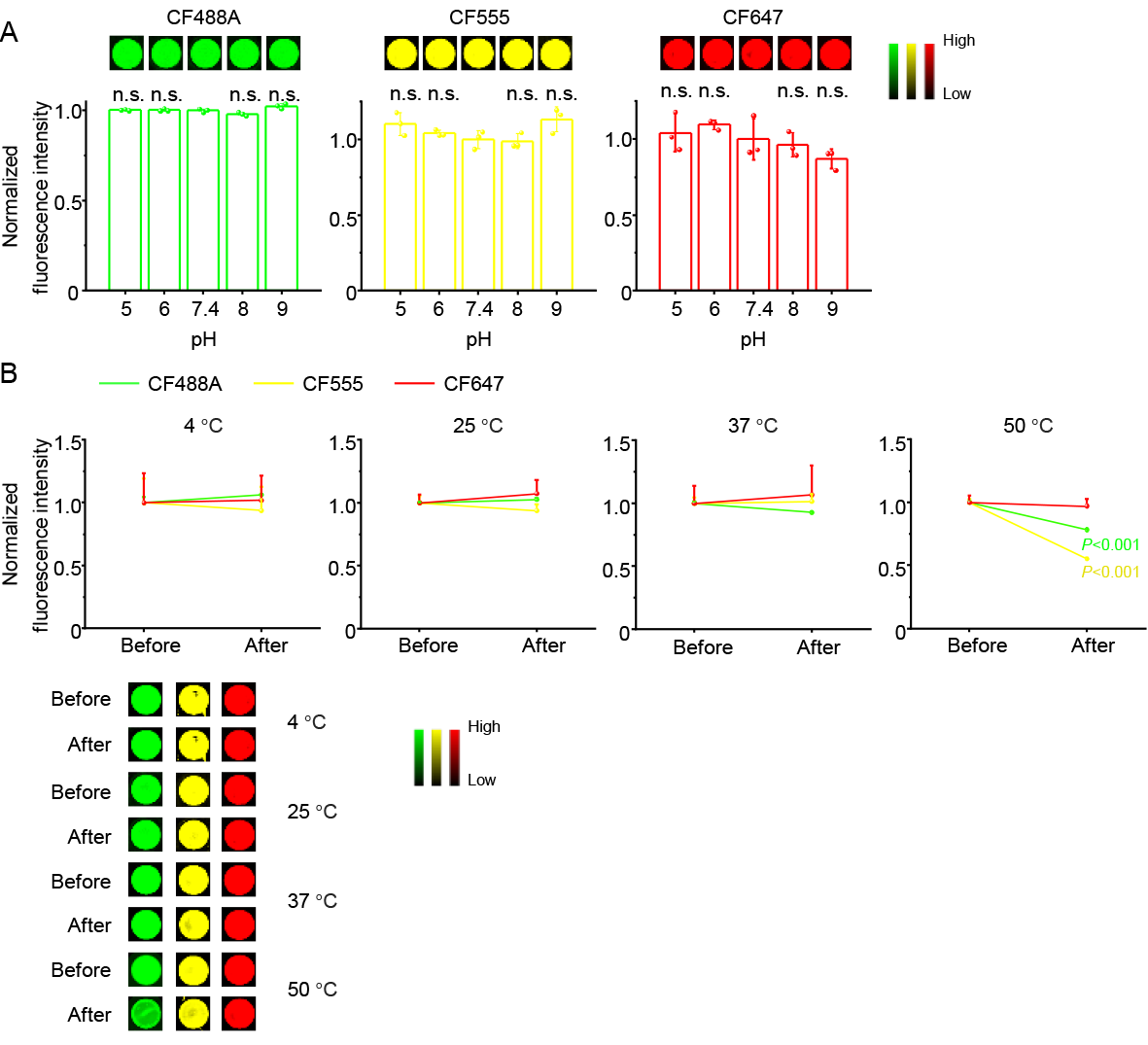


**Supplementary Fig. 24 | Effects of pH and temperature on fluorescence signals. (A)** Fluorescence intensities of 100 nM BSA-fluorophores in phosphate buffers of pH 5, 6, 7.4, 8, and 9 after 2 h incubation at room temperature on AgNIS. The fluorescence intensities were normalized by the averaged intensity at pH 7.4. Physiological pH ranging from 5-9 did not exhibit statistically significant changes on signals of all colors. **(B)** After incubation with 100 nM BSA-fluorophores, AgNIS was preserved at 4, 25, 37, and 50 °C for 1 h. The fluorescence intensities of CF488A and CF555 decreased significantly after incubation at 50 °C, while this high temperature did not affect CF647. In addition, all other temperatures exerted no effect on all signals. Since assays are usually carried out under ambient conditions of 25 °C and pH 7.4, we can conclude that neither pH nor temperature will significantly influence our assay under normal circumstances.

**Supplementary references:**

1. Tan YH, Liu M, Nolting B, Go JG, Gervay-Hague J, Liu GY. A nanoengineering approach for investigation and regulation of protein immobilization. *ACS Nano* **2**, 2374-2384 (2008).

2. Wright AK, Thompson MR. Hydrodynamic structure of bovine serum albumin determined by transient electric birefringence. *Biophys J* **15**, 137-141 (1975).

3. Hsu L-Y*, et al.* Large-scale inhomogeneous fluorescence plasmonic silver chips: origin and mechanism. *Chem* **6**, 3396-3408 (2020).

4. Kong J, Yu S. Fourier transform infrared spectroscopic analysis of protein secondary structures. *Acta Biochim Biophys Sin (Shanghai)* **39**, 549-559 (2007).

5. Du P, Zhao J, Mashayekhi H, Xing B. Adsorption of bovine serum albumin and lysozyme on functionalized carbon nanotubes. *J Phys Chem C* **118**, 22249-22257 (2014).
